# Toll-like receptor chaperone *HSP90B1* and the immune response to *Mycobacteria*

**DOI:** 10.1371/journal.pone.0208940

**Published:** 2018-12-14

**Authors:** Andrew D. Graustein, Elizabeth A. Misch, Munyaradzi Musvosvi, Muki Shey, Javeed A. Shah, Chetan Seshadri, Augustine Aguoju, Kathryn Bowman, Humphrey Mulenga, Ashley Veldsman, Willem A. Hanekom, Mark Hatherill, Thomas J. Scriba, Thomas R. Hawn

**Affiliations:** 1 University of Washington, Seattle, WA, United States of America; 2 South African Tuberculosis Vaccine Initiative, Institute of Infectious Disease & Molecular Medicine and Division of Immunology, Department of Pathology, University of Cape Town, Cape Town, South Africa; 3 Veterans Affairs Puget Sound Health Care System, Seattle, WA, United States of America; 4 Bill and Melinda Gates Foundation, Seattle, WA, United States of America; Institut de Pharmacologie et de Biologie Structurale, FRANCE

## Abstract

**Rationale:**

HSP90B1, also known as gp96, is a chaperone for multiple Toll-like receptors (TLRs) and is necessary for TLR-mediated inflammatory responses in murine myeloid cells. The molecule is also expressed in T-cells though its specific role is unknown. We hypothesized that human HSP90B1 regulates monocyte and T-cell responses to *Mycobacterium tuberculosis* (Mtb) and bacilli Calmette-Guerin (BCG) and that its variants are associated with susceptibility to TB disease.

**Methods:**

We screened 17 haplotype-tagging SNPs in the *HSP90B1* gene region for association with BCG-induced T-cell cytokine responses using both an *ex-vivo* whole blood assay (*N* = 295) and an intracellular cytokine staining assay (*N* = 180) on samples collected 10 weeks after birth. Using a case-control study design, we evaluated the same SNPs for association with TB disease in a South African pediatric cohort (*N* = 217 cases, 604 controls). A subset of these SNPs was evaluated for association with *HSP90B1* expression in human monocytes, monocyte-derived dendritic cells, and T-cells using RT-PCR. Lastly, we used CRISPR/Cas9 gene editing to knock down *HSP90B1* expression in a human monocyte cell line (U937). Knockdown and control cell lines were tested for TLR surface expression and control of Mtb replication.

**Results:**

We identified three SNPs, rs10507172, rs10507173 and rs1920413, that were associated with BCG-induced IL-2 secretion (*p* = 0.017 for rs10507172 and *p* = 0.03 for rs10507173 and rs1920413, Mann-Whitney, dominant model). SNPs rs10507172 and rs10507173 were associated with TB disease in an unadjusted analysis (*p* = 0.036 and 0.025, respectively, dominant model) that strengthened with sensitivity analysis of the definite TB cases, which included only those patients with microbiologically confirmed Mtb (*p* = 0.007 and 0.012, respectively). Knockdowns of *HSP90B1* in monocyte cell lines with CRISPR did not alter TLR2 surface expression nor influence Mtb replication relative to controls.

**Conclusion:**

Among infants, an *HSP90B1* gene-region variant is associated with BCG-induced IL-2 production and may be associated with protection from TB disease. *HSP90B1* knockdown in human monocyte-like cell lines did not influence TLR2 surface localization nor Mtb replication. Together, these data suggest that HSP90B1 regulates T-cell, but not monocyte, responses to mycobacteria in humans.

## Introduction

*Mycobacterium tuberculosis* (Mtb) is one of the leading infectious causes of morbidity and mortality worldwide, with an estimated 8.6 million incident cases of tuberculosis (TB) and 1.3 million deaths annually [1]. While vaccination with *Mycobacterium bovis* Bacillus Calmette-Guerin (BCG) confers partial protection (particularly to children), its overall efficacy is not adequate for disease control [2]. The development of a more effective vaccine will be facilitated by a better understanding of the host response to Mtb infection.

Evidence from twin comparisons, genome wide linkage studies, and candidate gene studies suggest that host genetic factors play an important role in susceptibility to Mtb [3–5]. The human Toll-like receptors (TLRs) are a family of receptors that recognize pathogen-associated molecular patterns (PAMPs). Single nucleotide polymorphisms (SNPs) in *TLR* genes and in those genes encoding the intracellular signaling molecules associated with TLR activation are associated with susceptibility to infection in general as well as to TB disease specifically [6–11]. The molecular mechanisms behind these associations are not well understood.

The TLR signaling pathways require a complex set of intracellular molecular interactions not only for signal transduction but also for the biosynthesis, folding, and localization of the TLR molecules [12]. Heat shock protein 90β1 (HSP90B1, also known as GP96 in humans and GRP94 in mice) is a member of the heat shock protein 90 family [13] that has been identified as a chaperone for multiple client proteins, including many of the TLRs [14–16]. HSP90B1 primarily localizes to the endoplasmic reticulum [17]. While HSP90B1 can bind to many different client peptides, in mice it has been shown to be necessary for the chaperoning of most TLRs and some integrins [15], the canonical wingless/integrated (WNT) pathway coreceptor low-density lipoprotein receptor-related protein 6 (LRP6) [16], glycoproteins A receptor predominant (GARP) in T-cells [18], and the platelet glycoprotein Ib/IX/V complex [19]. The conditional knockout of *Hsp90b1* in a murine macrophage line led to a loss of tumor necrosis factor (TNF) and interleukin 6 (IL-6) responses to ligands for TLR2, TLR4, TLR5, TLR7, and TLR9 [20]. This result was corroborated in a study that identified an HSP90B1 co-chaperone, canopy3 (CNPY3), that was necessary for an inflammatory response to TLR2, TLR4, TLR7, and TLR9 ligands in mice [14]. Conditional *Hsp90b1* knockdown in murine macrophages also reduced phagocytosis of *Klebsiella pneumoniae* and decreased survival from *K*.*pneumoniae* infection [21]. While these lines of evidence support a role for this molecule in the murine innate immune response, little is known about the relationship between HSP90B1, TLRs, and innate immunity in humans.

Relative to the innate immune response, even less is known about the function of HSP90B1 in the adaptive immune response. In humans, HSP90B1 was identified as a component of the *Lat* interactome, which is responsible for the amplification and diversification of T-cell receptor signaling [22], though the role for the chaperone in this complex has not been determined. Conditional knockout of *Hsp90b1* in Treg cells resulted in an uncontrolled autoimmune inflammatory response in mice and a loss of the Treg phenotype [23]. Used as a vaccine adjuvant to a BCG vaccine, HSP90B1 was recently shown to enhance T-cell responses and protection from *M*. *bovis* in mice [24]. We do not know if HSP90B1 influences T-cell responses indirectly as a chaperone for innate immune molecules or directly as a chaperone of T-cell signaling molecules.

In this paper, we examine the role of *HSP90B1* genetic variants on BCG-induced T-cell cytokine responses and the development of TB disease in a South African pediatric cohort. We hypothesized that genetic variants would influence both monocyte and T-cell mediated immune responses to Mycobacteria and protection from TB disease.

## Materials and methods

### Research/Study participant recruitment

Study participants were enrolled by the South African Tuberculosis Vaccine Initiative (SATVI) at field sites in Worcester, South Africa [11, 25–28]. This region has one of the highest incidences of pediatric TB disease in the world [29]. The cohort used in the current study is part of a larger BCG vaccination correlates-of-risk project with 11,680 infants and has been described in detail previously [25, 28, 29]. Enrolled infants were vaccinated with BCG at birth, as is standard practice in South Africa. A nested genetics case-control study was performed to identify cases and controls during a 2-year prospective observation period as previously described [30]. The cases and controls included those from Cape Mixed Ancestry (CMA) and Black African descent. The CMA ethnicity represents the genetic admixture of Khoisan, Black African, European and both east and south Asian populations that has existed for over 350 years [31, 32]. Several terms have been used to describe CMA including South African Mixed Ancestry and “Coloured”, a collective term for people of mixed ancestry in southern Africa, which is an officially recognized census term in South Africa and routinely used for self-classification.

The criteria for the case and controls definitions have been described previously [28, 29] and are summarized below. Community-wide passive surveillance systems identified patients with TB disease and children with symptoms suggestive of TB disease. Briefly, all children with symptoms consistent with TB disease or who had contact with an adult with TB disease were admitted to a dedicated research ward for examination, chest imaging, tuberculin skin testing, two early-morning gastric aspirates, and two induced sputa for Mtb smear and culture. Subjects were described as “definite TB” if they had a positive Mtb culture, smear, or PCR from one of their samples. Subjects were described as “probable TB” if they had a chest radiograph consistent with or suggestive of TB in addition to one or more laboratory or clinical features (smear negative, cough >2 weeks, PPD skin test ≥ 15mm, failure to thrive, and recent weight loss). Subjects diagnosed with TB by the treating physician and with 2 or more clinical features suggestive of TB but without consistent chest radiography were described as “possible TB”. Subjects with a symptom consistent with TB but with no other supporting evidence were labeled “unlikely TB”. All others were described as “not TB”. Two types of controls were enrolled. Household controls were children without TB disease over a 2 year follow up period who were living with an individual with active TB disease. Community controls had no history of TB disease in first two years of life. Demographic information for the cohort is described in the supporting information (S1 Table).

Exclusion criteria included HIV positive infant or mother, BCG vaccine not administered within 24 hours of birth, significant perinatal complications in the infant, any pre-existing acute or chronic disease in the infant, or clinically apparent anemia. Parents or legal guardians of study participants were informed of the risks and benefits of study participation and signed informed consent prior to enrollment. The protocol was approved by the University of Cape Town Research Ethics Committee, the Aeras Global TB Vaccine Foundation, and the University of Washington Institutional Review Board. Two audits were done during the enrollment period, on behalf of the sponsor, by an independent contract research organization, Triclinium, Johannesburg, South Africa [29].

### SNP selection

**Fig 1:**
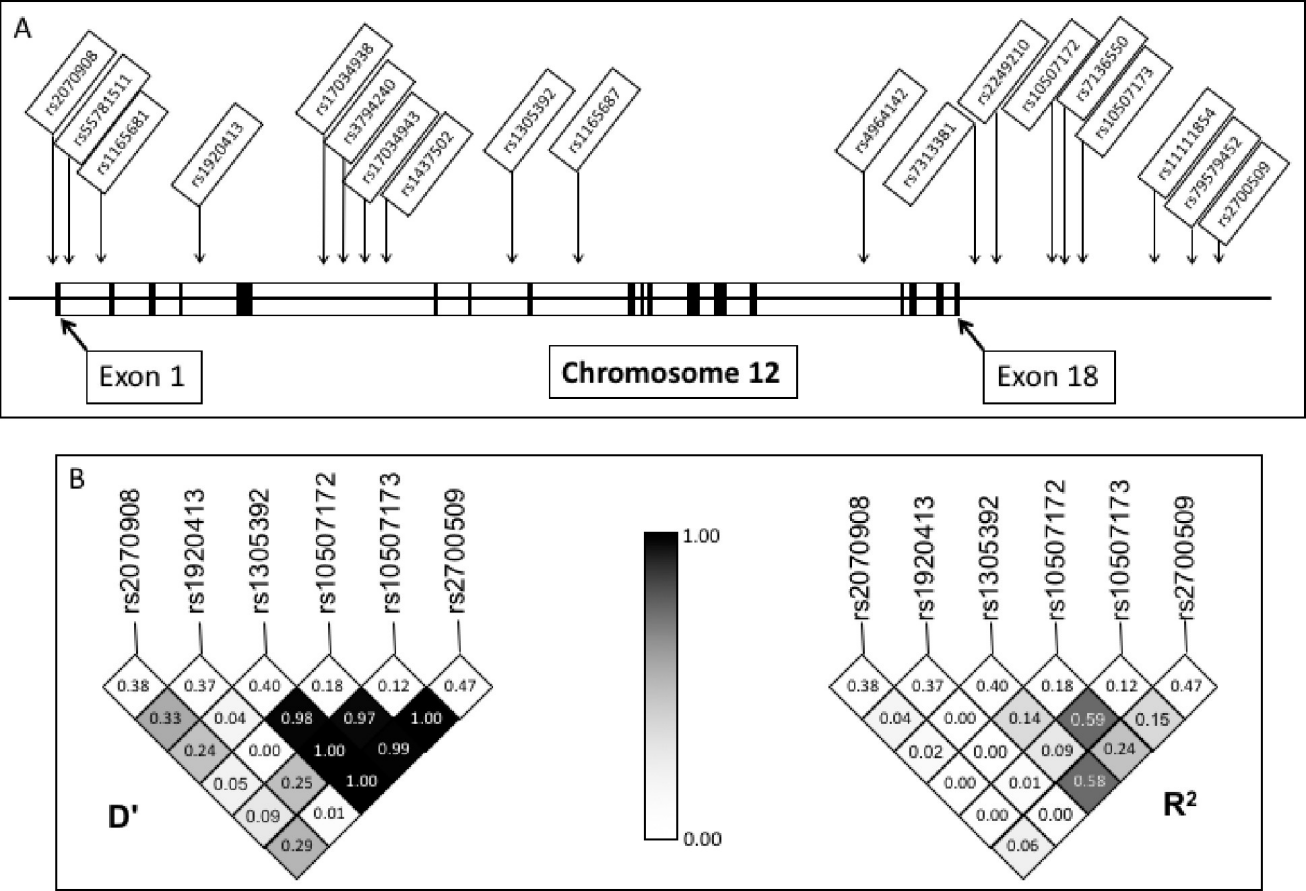
SNP selection and linkage disequilibrium. Haplotype-tagging SNPs included in our study are shown with their location relative to the *HSP90B1* gene (A). Measures of linkage disequilibrium by D’ and R^2^ are shown for selected SNPs (B).

We identified haplotype-tagging SNPs from the International HapMap Project (http://www.hapmap.org) and other public databases within the Genome Variation Server (http://www.ncbi.nlm.nih.gov.offcampus.lib.washington.edu/SNP/ and www.innateimmunity.net). We searched a region on chromosome twelve ten kilobases upstream and downstream of the *HSP90B1* gene as well as within the gene itself for tagged SNPs in the Yoruba in Ibadan population (YRI) using an R^2^ cutoff of 0.8 for linkage disequilibrium and a minor allele frequency cut-off of 5%. We used STATA version 14 and the software program *PWLD* (StataCorp, College Station, TX) to calculate R^2^ and D’ as measurements of linkage disequilibrium between polymorphisms. Using the above criteria, we identified 19 SNPs for analysis (Fig 1). Hardy-Weinberg equilibrium was tested for each SNP in the CMA control population. Two of these SNPs, rs17034939 and rs7313381, were in violation of Hardy-Weinberg equilibrium (x^2^ *p<*0.001) and were not analyzed further. Chromosome location and genotypic and allelic frequencies for each SNP in the case and control populations are described in the supporting information (S2 Table) as are linkage disequilibrium R^2^ values from the CMA control group (S3 Table). For reference, genotype and allele frequencies for the YRI population are described the supporting information (S4 Table) as are linkage disequilibrium R^2^ values for select SNPs in the YRI population (S5 Table). For the case-control study we also included a set of 96 Ancestry Informative Markers (AIMS) for the CMA population. Further details of this analysis are provided in the Statistical Analysis section of the Materials and Methods section and in the supporting information (S6 Table).

### Genotyping

Genomic DNA was prepared from peripheral blood using the QIAmp DNA Blood or Blood and Tissue Kit (Qiagen). Genotyping was performed using an Illumina GoldenGate genotyping platform (Illumina, Inc) or a Fluidigm array platform (Fluidigm Corporation) and a subset of calls for one SNP (rs10507173) were genotyped using the TaqMan SNP Genotyping Assay (Applied Biosystems, Step One Plus instrument) to confirm genotyping accuracy.

### Cytokine assays

Whole blood cytokine levels and intracellular cytokine staining were conducted on heparinized blood drawn from 10-week old South African infants. 10 milliliters of blood were taken and used both for the cytokine assays and genotyping. Samples were stimulated *ex-vivo* with media or BCG (strain SSI, 1.2 x 10^6^ CFU’s/ml) for 7 hours at 37°C. For whole-blood assays, IL-2 and IFN- γ were measured by multiplex bead array technology according to manufacturer’s instructions (Bio-Rad, Hercules, CA, USA) and read on a Luminex luminometer (Luminex, Austin, TX, USA). Basal cytokine levels measured in plasma harvested from unstimulated blood were subtracted from values obtained from BCG-stimulated blood [11].

For intracellular cytokine staining, Brefeldin A was added to the above samples and cells were incubated for 5 additional hours. Samples were frozen in 40% RPMI and 40% DMSO with 10% FCS and thawed prior to the time of data analysis. Flow cytometry was performed at the University of Washington Flow Cytometry Core Facility on an LSRII 5-laser flow cytometer (Becton Dickenson, Inc) using antibodies against cell surface markers including: anti-CD3 ECD (Beckman Coulter, UCHT1), anti-CD4 APC-Alexa Fluor 750 (Beckman Coulter, 13B8.2), and anti-CD8 PerCP Cy5.5 (BD, SK1). Antibodies against cytokines included anti-IL-2 PE (BD, MQ1-17H12) and anti-IFN-γ (BD, B27) [33].

### *HSP90B1* gene expression in leukocytes

Peripheral blood mononuclear cells (PBMC’s) were isolated from whole blood using Ficoll gradient separation. Monocytes, dendritic cells (DC’s), and T-cells were isolated as described below for each cell type. Monocyte samples were derived from PBMC’s from South African donors. DC’s and T-cells were derived from healthy donors local to Seattle, WA. Sixty milliliters of blood were taken from the local, healthy cohort for genotyping and cell isolations. Following cell isolation, RNA was collected using the RNeasy kit (Qiagen). Single-stranded cDNA was generated from RNA samples using Multiscribe reverse transcriptase (Invitrogen). Real-time PCR was performed on an Applied Biosystems One Step Plus Real Time PCR System (ABI) using *HSP90B1* probe Hs.PT.53a.4251075 (Integrated DNA Technologies) and GAPDH-JOE (ABI 402869).

#### Monocytes

PBMCs were thawed and plated to tissue culture plates overnight in the presence of 50ng/ml M-CSF with RPMI supplemented with 10% FCS. RNA was isolated from plate-adherent monocytes using RNeasy kits (QIAGEN, Inc). Nonadherent cells were used to collect genomic DNA using peripheral blood DNA collection kits (QIAGEN). RNA concentrations were determined using Nanodrop; cDNA was amplified for long-term storage at -20C. qPCR studies were performed on an ABI Step One Plus (ABI, Inc). For gene expression assays following LPS stimulation, monocytes were exposed to media for four hours. Cells were subsequently harvested for RNA collection and RT-PCR as described above.

#### Dendritic cells

Dendritic cells were generated from monocytes and confirmed by flow cytometry as previously described [34]. Briefly, monocytes were isolated from PBMCs by positive selection using CD14 microbeads (Miltenyi Biotec) and magnetic column separation. Monocytes were then incubated in RPMI + 10% FCS (Life Technologies) supplemented with L-glutamine and GM-CSF (100ng/ml) plus IL-4 (20ng/ml) (PeproTech) for 3 days.

#### T-cells

T-cells were isolated from PBMCs by positive selection using CD3 microbeads (Miltenyi Biotec) and magnetic column cell separation. T-cell survival and specificity was confirmed by flow cytometry with live/dead stain aqua (Life Technologies) and CD3-ECD (Beckman-Coulter).

### CRISPR/Cas-9 knockdown generation

We used a lentiviral delivery system in human monocyte-like cell lines to generate CRISPR/Cas-9 knockdowns of the *HSP90B1* gene as previously described [35]. Guide RNA targeting *HSP90B1* (sense sequence: AAA GGA CGA AAC ACC GGC ACG CCA TGA GGG CCC TGG TTT TAG AGC TAG AAA TAG CAA G; antisense sequence: CTT GCT ATT TCT AGC TCT AAA ACC AGG GCC CTC ATG GCG TGC CGG TGT TTC GTC CTT T) was selected using ChopChop (http://chopchop.cbu.uib.no/). Guide RNA and scaffold were cloned into a lentiviral plasmid, gRNA-Cas9-t2a-puromycin pRRL (gift from the laboratory of Dan Stetson, University of Washington), using Clontech InFusion Cloning Kit (Takara Bio). Nontargeting control gRNA-Cas9-t2a-puromycin pRRL plasmids were used as negative controls (sequences were obtained from the GeCKO v2 library, Addgene) [36]. Lentivirus was produced in HEK 293T Lenti-X cells (Clontech) by transfecting HSP90B1-pRRL plasmid with packaging plasmids pRSV-Rev, pMD2.g, and pMDLg/pRRE (Addgene numbers 12253, 12259, and 12251, respectively), in Opti-MEM and TransiT-LT1 (Mirius Bio). Supernatants were filtered and incubated with U937 (ATCC CRL-1593.2) cells and 5ug/mL polybrene (EMD Millipore). Transduced U937 cells were rested for 24 hours in fresh media after incubation with lentiviral particles. Transductants were selected with puromycin. CRISPR/Cas-9 targeting was evaluated by PCR amplification of genomic DNA containing the target site followed by restriction fragment-length polymorphism (RFLP) screening for the loss of an *ApaI* restriction site within the Cas-9 cut site. HSP90B1 protein production was evaluated using mouse anti-Grp94 antibody (Cell Signaling 2104) with anti-beta actin as a loading control (Cell Signaling 8457).

### TLR2 surface expression

Wild type and *HSP90B1* knockdown U937 cell lines were incubated with either mouse anti human TLR2 conjugated to APC (eBioscience 17–9922) or isotype control. Flow cytometry was performed at the University of Washington Flow Cytometry Core Facility on an LSRII 5-laser flow cytometer (Becton Dickenson, Inc).

### *Mtb* replication assay

U937 cell lines containing the *HSP90B1* knockdown or the empty vector or wild type controls were plated in sextuplet at a density of 75,000 cells/well and differentiated with PMA at 50ng/ml overnight, washed x 2 with HBSS, and rested for 24 hours. A frozen aliquot of Mtb Erdman strain expressing lux (Mtb-lux, a gift from Jeffrey Cox, University of California, San Francisco) was thawed, centrifuged, and resuspended in RPMI with 10% FBS. Luminescence (relative light units, RLU’s) are a proxy for colony count using this assay [30]. To act as a control for background luminescence, a separate set of wells had Mtb-Lux added without human cells. Relative light units were measured daily for 1 week following infection on a plate reader (Synergy H4 reader, BioTek Instruments). Means of daily luminescence values, standard deviations, and two-tailed Student’s *t* tests were performed and graphed using GraphPad Prism Version 7.0.

### Statistical analysis

We used STATA 14 (StataCorp) to perform Pearson’s *X*^*2*^ testing and logistic regression on the case-control cohort and general linearized modeling or analysis of variance for cytokine assays and gene-expression assays, as specified in the text for each experiment. We used Prism 7 (GraphPad Software, Inc) for analysis of normality and nonparametric testing for cytokine assays. Results were not adjusted for multiple comparisons. For the case-control study, to control for potential genetic heterogeneity in the CMA ethnicity, we included a set of 96 Ancestry Informative Markers (AIMS) for the complex five-way admixed South African Coloured population as described previously [28, 30, 37]; see S6 Table for genotype and allele frequencies of AIMs SNPs in the case and control populations. In CMA individuals, there were no significant differences in genotype frequencies of the AIMS between cases and controls. The AIMS were used to calculate a coefficient incorporating the first five principal components of the AIMS data, which accounted for over 60% of the variation in the dataset. We then used this data to create a regression coefficient for adjusting the primary case-control data for ethnicity by converting it into an ethnicity principal component coefficient using the “pca” command in STATA 14. This provided an alternative means of regressing for ethnicity within the CMA populations.

## Results

### Association of *HSP90B1* gene region SNPs with BCG-induced cytokine responses in South Africa

**Fig 2:**
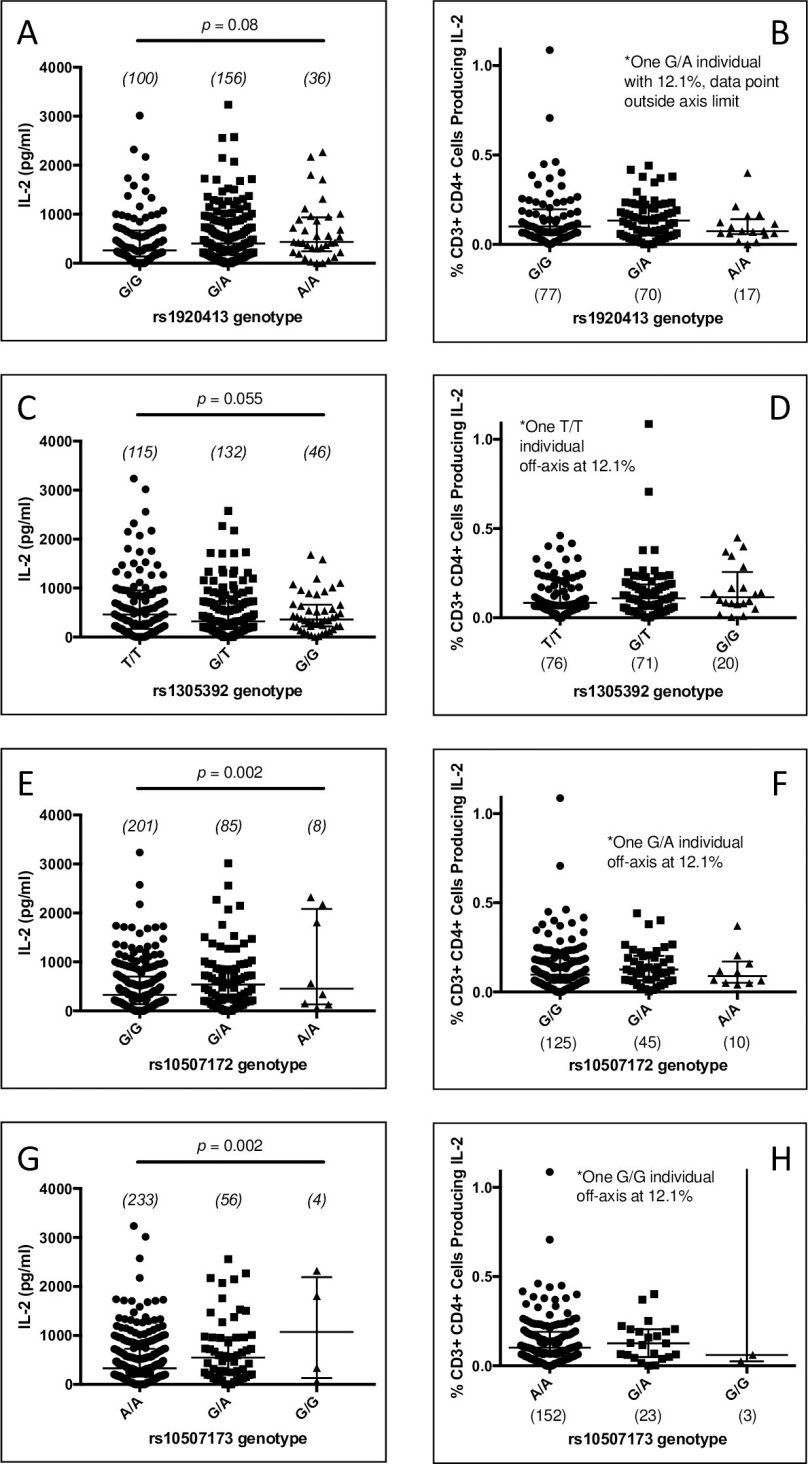
BCG-induced whole blood and intracellular cytokines. Blood drawn from 10-week old infants was stimulated *ex-vivo* with BCG. For whole-blood secreted cytokines, (A, C, E, G), IL-2 and IFN- γ supernatant levels were measured by enzyme-linked immunosorbent assay. Median and interquartile ranges of stimulated (BCG) minus unstimulated levels are shown for selected SNPs (with p-values calculated by a generalized linear model). Genotype frequencies are provided in parentheses above each genotype. For intracellular cytokine assays (B, D, F, H), an independent sample set was stimulated with BCG as above and brefeldin A added 7 hours later. Samples were evaluated using flow cytometry for cell surface markers and for intracellular cytokines IL-2 and IFN- γ. Median and interquartile ranges are shown for CD4+ T-cells for select SNPs. Genotype frequencies are provided in parentheses below each genotype. H: *p* = 0.0001 (analysis of variance (ANOVA))), *p* = 0.59 without outlier. *p*-values nonsignificant for B, D, and H (ANOVA).

To examine the role of *HSP90B1* in TB pathogenesis, we assessed whether genetic variants are associated with innate and adaptive immune responses as well as clinical disease. Following BCG vaccination at birth in South Africa, infant blood was drawn at 10 weeks of age and re-stimulated with BCG. We measured secretion of IL-2 and IFN-γ [29]. To assess T-cell responses, we first examined whether 17 haplotype-tagging *HSP90B1* gene region SNPs were associated with whole blood secreted cytokine responses to *ex-vivo* BCG stimulation (N = 295) (Figs 1 and 2, S7 Table).

SNPs rs10507172 and rs10507173 were associated with IL-2 production (*p* = 0.002) while two other SNPs (rs1305392 and rs1920413) showed a trend towards association with IL-2 production (*p* = 0.055 and 0.080, respectively) (general linearized model, Fig 2A, 2C, 2E and 2G). No other SNPs were associated with IL-2 secretion nor with secretion of IFN-γ (S7 Table). Due to low numbers of minor-allele homozygotes for rs10507172 and 105017173, we examined a dominant model which showed that rs10507172, rs10507173 and rs1920413 were associated with IL-2 production (p = 0.017, 0.03, and 0.03, respectively, Mann-Whitney). In each case the minor allele was associated with an increase in IL-2 production. rs10507172 and rs10507173 were in moderate linkage disequilibrium (R^2^ = 0.59, D’ = 0.97, Fig 1). Together, these data suggest that HSP90B1 genetic variants may be associated with BCG-specific T-cell responses.

To determine a cellular origin of HSP90B1-associated T cell responses, we performed intracellular cytokine staining with flow cytometry to evaluate for BCG-specific IFN-γ or IL-2 CD4+ or CD8+ T cell memory responses in an independent set of samples (N = 180). Of the four SNPs with significant or borderline significant IL-2 associations in the whole blood assay, only rs10507173 was associated with CD4+ IL-2 production (*p*<0.0001, general linearized model), though the association was driven by a single minor allele homozygote outlier (2H) and was not significant under a dominant model (*p* = 0.95). None of the other SNPs were associated with IL-2 or with IFN-γ in CD4+ cells, nor with the same cytokines in CD8+ cells (S8 Table). Together, these data are inconclusive but suggest that rs10507173 may be associated with BCG-induced IL-2 produced by CD4+ T cells.

### *HSP90B1* gene region SNPs are not associated with gene expression levels in monocytes, dendritic cells, or T-cells

SNPs rs10507172, rs10507173, rs1920413, and rs1305392 fall in noncoding sequence either within intronic regions (rs1920413, rs1305392) or in the 3’ downstream region of *HSP90B1* (rs10507172, rs10507173) (Fig 1). To explore the mechanisms by which these SNPs may influence IL-2 cytokine production and pediatric TB disease, we evaluated whether any polymorphisms were expression quantitative trait loci (eQTLs).

**Fig 3:**
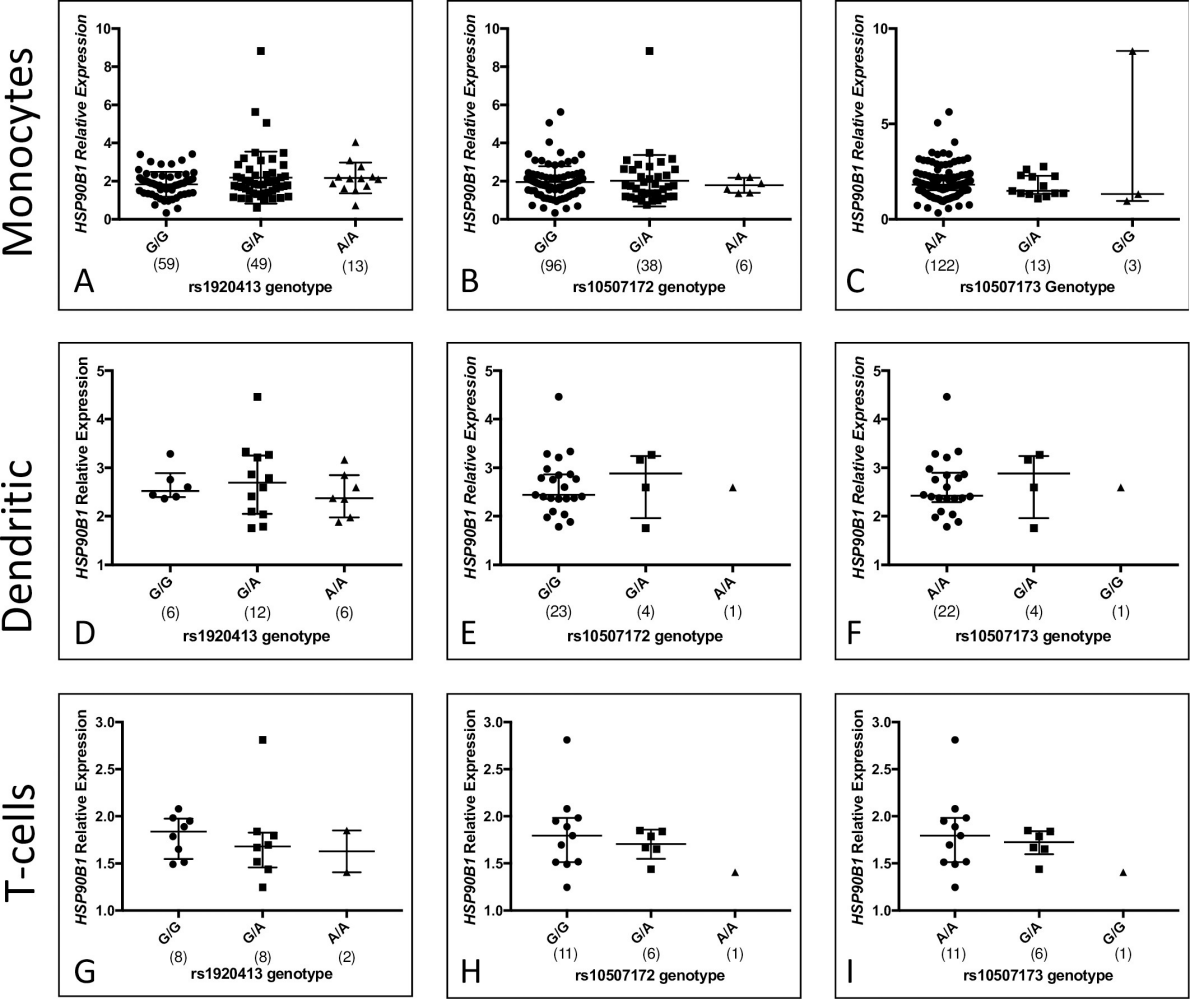
*HSP90B1 variants are not associated with* gene expression. *HSP90B1* expression was compared to expression of GAPDH in unstimulated monocytes (A, B, C), dendritic cells (D, E, F), and T-cells (G, H, I) and stratified by genotype. Median and interquartile ranges are included and sample sizes are provided in parentheses below each genotype. Results for select SNPs are shown. Results were not significant for any of the SNPs evaluated (*p*>0.05, ANOVA).

We examined whether HSP90B1 SNPs were associated with *HSP90B1* gene expression in monocytes from the same South African cohort (Fig 3A, 3B and 3C). The rs10507173 rare variant was associated with increased expression (*p* = 0.007, ANOVA). However, a single outlier homozygous for the rare variant contributed to the association and, in the absence of the outlier, an association was not seen. We did not find an association between rs10507172, rs1920419 or rs1305392 genotype and gene expression in monocytes (graphs for rs1305392 shown in S1 Fig). We did not identify an association between genotype in rs10507172, rs10507173, rs1920419, or rs13105392 and *HSP90B1* expression in either monocyte-derived DCs or T-cells. (Fig 3D–3I). Together, these data suggest that HSP90B1 variants are not eQTLs in monocytes, dendritic cells, or T cells.

### *HSP90B1* knockdown did not influence TLR2 surface expression or Mtb replication in a monocyte cell line

**Fig 4:**
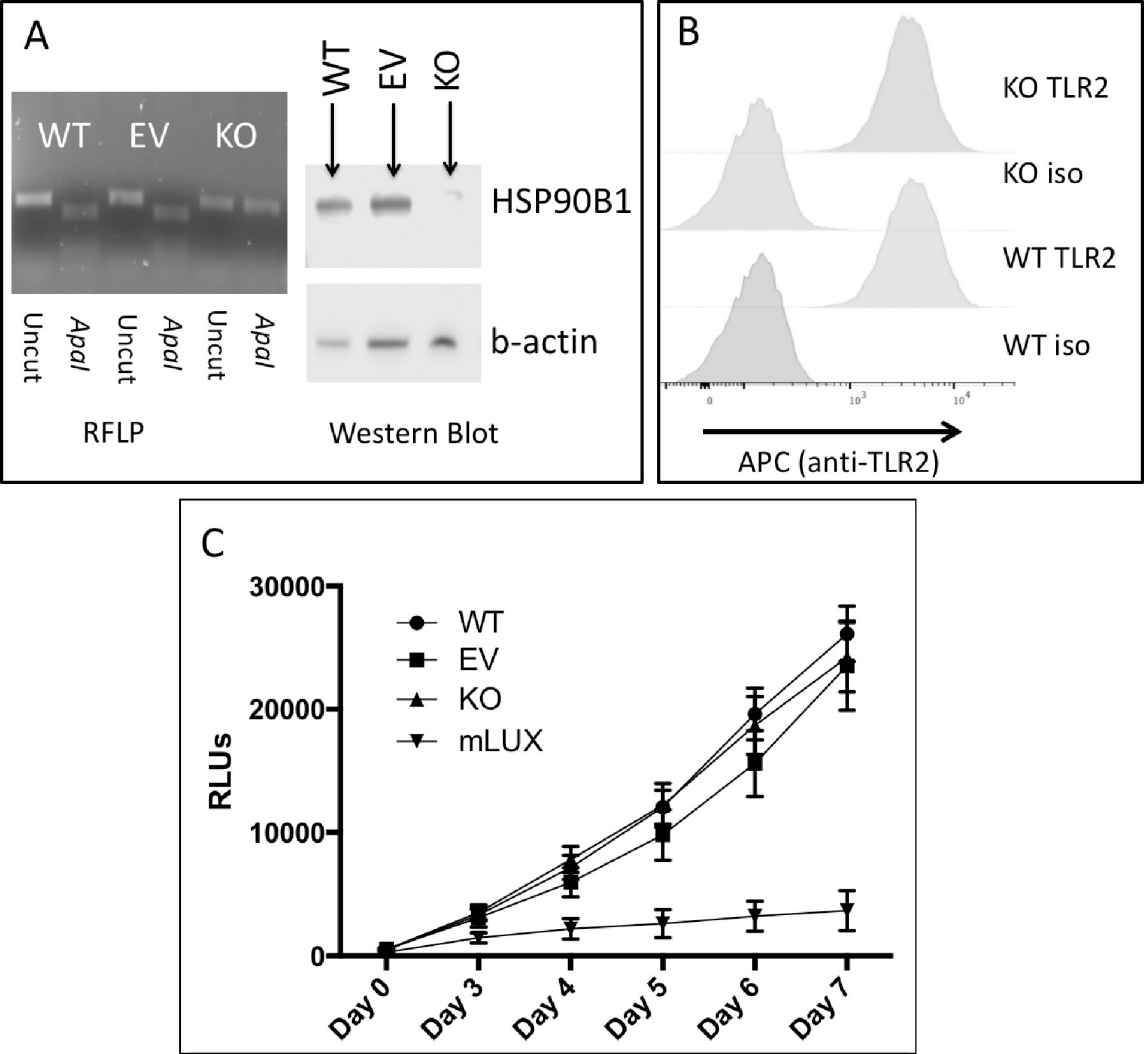
*HSP90B1* knock down does not alter TLR2 surface expression or Mtb replication. Using CRISPR/Cas9, *HSP90B1* was targeted for gene deletion in U937 macrophage-like human cells. **A:** Confirmation of knockout. Restriction fragment length polymorphism (RFLP) verified the loss of the *ApaI* restriction endonuclease site in the targeted lines (KO) but not the wild type (WT) nor cell line exposed to the CRISPR apparatus with a nonspecific guide sequence (EV). Western blot revealed reduction of the expected band in the KO line (b-actin as loading control). **B:** Wildtype or *HSP90B1* knock down cell lines were stained with anti-TLR2 antibody or isotype control (iso) and evaluated for surface expression using flow cytometry. **C:** Knock down or control cell lines were infected with Erdman strain Mtb constitutively expressing luciferase at a multiplicity of infection of 5. Relative light units (RLUs) act as a proxy for colony count in this assay. Daily RLUs were measured from day 3 to day 7. Included is a background control where the Mtb was added to wells in the absence of human cells (mLux). Each exposure was repeated in sextuplet and the mean and standard deviation are provided for each cell line and time point.

We next examined whether HSP90B1 mediated antimicrobial effects in Mtb-infected macrophages. Using CRISPR/Cas-9 technology we targeted early exonic regions of the *HSP90B1* gene for knockout in the monocyte-like U937 cell line (Fig 4A). Western blot confirmed at least partial knockdown of HSP90B1, although an HSP90B1 band at ~100 kDa remained after CRISPR/Cas9 targeting and suggested possible partial expression of full length HSP90B1. Since previous studies have shown that *Hsp90b1* is necessary for surface TLR expression and TLR-mediated signaling in mice, we tested the knockdown monocyte line for surface expression of TLR2 but did not observe a difference between KO and control cell lines (Fig 4B). We also did not see a difference in Mtb intracellular replication between the *HSP90B1* knockdown and the control lines (Fig 4C). Together, these data suggest that HSP90B1 does not regulate TLR2 surface expression or bacterial growth in monocyte cell lines infected with Mtb.

### Association of *HSP90B1* gene region SNPs with TB disease in South African children

To test the clinical significance of the association of *HSP90B1* variants with BCG-induced T-cell responses, we used a case-control study design in a South African pediatric cohort to determine if *HSP90B1* gene-region SNPs are associated with pediatric TB disease. We genotyped *HSP90B1* SNPs in 217 cases of children with TB disease and 604 controls (Table 1, S1 and S2 Tables). SNPs rs10507172, rs10507173, and rs1920413 showed significant or near-significant associations with TB disease using a dominant model in an unadjusted analysis (*i*.*e*. comparing AA vs Aa/aa; *p* = 0.036, *p* = 0.025, and *p* = 0.069, respectively). When adjusting for gender, all three SNPs again showed significance or near-significance (OR = 0.68, *p* = 0.035 for rs10507172, OR = 0.62, *p* = 0.023 for rs10507173 and OR = 0.74, *p* = 0.063 for rs1920413). All three SNPs have an odds ratio less than one, suggesting that the minor allele is protective from the development of pediatric TB disease. SNP rs1305392, which was also borderline associated with IL-2 production, was not significantly associated with TB disease.

**Table 1:** *HSP90B1* SNPs and TB disease in a South African pediatric cohort.

		*A/A*	*A/a*	*a/a*	*Genotypic*		*Dominant*		
*SNP*	*Group*	*N (%)*	*N (%)*	*N (%)*	*x2*	*p*	*p*	*OR 95% CI*	
rs1920413	Control	222 (37)	306 (51)	75 (12)					
	TB	95 (44)	95 (44)	25 (12)	3.6	0.17	0.069	0.75	0.54–1.02
rs1305392	Control	227 (38)	271 (45)	105 (17)					
	TB	71 (33)	103 (48)	43 (20)	1.8	0.41	0.196	1.2	0.89–1.72
rs10507172	Control	406 (67)	179 (30)	17 (3)					
	TB	163 (75)	49 (23)	5 (2)	4.4	0.11	0.036	0.69	0.48–0.98
rs10507173	Control	467 (78)	128 (21)	7 (1)					
	TB	184 (85)	31 (14)	2 (1)	5.1	0.077	0.025	0.62	0.41–0.94

Results of the Pearson’s *x*^2^ test for the genotypic and dominant model are shown for select SNPs along with the odds ratio (*OR*) and 95% confidence interval (*CI*) for the dominant model. Sample size (*N*) and genotype frequency (%) are shown for each genotype. Percentages are rounded to the nearest whole value, causing the sum of the genotype frequencies for some SNPs to not equal 100%. Detailed genotype and allele frequencies are provided in S2 Table.

We analyzed for strength of association in each ethnic population alone (S9 Table) or adjusted for population admixture with ancestry informative markers and found the same directions of effect, though less statistical significance (*p* = 0.29, OR = 0.78 for rs10507172, *p* = 0.134, OR = 0.65 for rs10507173 and *p* = 0.177, OR = 0.75 for rs1920413 under dominant models adjusting for AIMs; S10 Table). Finally, we performed a sensitivity analysis by examining the association based on the strength of the clinical case classifications (N = 68 Definite TB, 111 Probable TB and 38 Possible TB with genotyping data for these SNPs). When only Definite and Probable cases were included, the association was stronger for all three SNPs (*p* = 0.007, OR = 0.58 for rs10507172, *p* = 0.012, OR 0.55 for rs10507173 and *p* = 0.051, OR 0.71 for rs1920413; S11 Table). Together, these data suggest a possible association of three HSP90B1 SNPs with a decreased risk of pediatric TB.

## Discussion

Our primary findings suggest that three *HSP90B1* gene region SNPs are associated with BCG-specific IL-2 in T-cells in a South African pediatric cohort and that another SNP had a trend toward association. Two of these SNPs were associated with TB disease in an unadjusted analysis and the association strengthened using the most strict diagnostic criteria. We found no evidence that HSP90B1 regulated monocyte responses to Mtb.

The finding of increased IL-2 and protection from TB disease is consistent with a model in which an enhanced Th1 response is protective [38], though we might also expect an increase in IFN-γ, which we did not see. IL-2 expression by T cells is a feature of central memory T-cells while IFN-γ expression, especially in the absence of IL-2, is a feature of effector or effector memory T-cells [39].

Our study has several limitations and strengths. First, the BCG-specific T-cells associations described above in the discovery sample set, when corrected for multiple comparisons, do not meet a significance threshold of *p* < 0.05. Using a conservative Bonferroni approach and considering that 17 SNPs were included in the analysis, we would require a threshold of *p* = 0.0029 (0.05/17) to achieve significance. Second, the TB case-control data became more highly significant when using strict criteria for the diagnosis of TB disease. This observation may be due to misclassification of the less stringent cases of ‘probable TB’ that were based on symptoms and chest x-ray findings. Third, pediatric TB is paucibacillary and difficult to diagnose. This challenge requires classifying case likelihood by different criteria. Reassuringly, the sensitivity analysis suggested a similar magnitude of effect in the definite TB cases. Despite these challenges, this pediatric cohort is one of the largest available in the world for genetic studies and presents opportunities for genetic insights that may be fundamentally different than adult TB.

Our negative results with the CRISPR knockdown in monocytes are surprising, particularly given the dramatic loss of TLR-mediated signaling seen in mouse conditional knockouts. One limitation to our innate immune molecular findings includes the possibility of an incomplete knockout of the molecule. While western blotting showed clear evidence of a reduction in expressed protein, a band roughly corresponding to the expected molecular weight of HSP90B1 was present in the knockout. In a mouse model showing loss of TLR signaling in the absence of the protein, *Hsp90B1* was completely knocked out using CRE/Flox in conditional macrophage lines [20]. It is possible that even a small amount of functioning molecule, for example the approximate 1/10 band density seen in our U937 knockdown line, is sufficient to chaperone TLRs and thus produce no appreciable drop in surface expression.

The association of HSP90B1 SNPs with BCG-specific T-cell responses suggests a possible direct regulatory role on T-cell function. *HSP90B1* is expressed in T-cells and may play an as-yet undetermined role in the downstream signal amplification of the T-cell receptor [22]. Interestingly, Foxp3-selective conditional knockout of *Hsp90B1* in murine T-cells led to a loss of Treg phenotype and an uncontrolled inflammatory response which included increased IL-2 production [23]. These data provide a possible Treg-dependent model for HSP90B1 regulation of BCG-specific T-cell responses. This is supported by evidence that GARP, a molecule that may also be chaperoned by HSP90B1, is a marker of activated FOXP3-expressing Tregs in humans [40].

Both historic and recent evidence describes a potential role for HSP90B1 as a vaccine adjuvant [24, 38, 41]. These studies have focused on the use of the molecule provided via an exogenous route and also in combination with a vaccine such as BCG. In this paper we studied the endogenous role of the molecule, specifically with regards to its function as a chaperone for TLRs. Whether there is a link between the endogenous function of HSP90B1 and its activity as an adjuvant remains to be seen. One possibility is that client-bound HSP90B1 is exposed to other immune cells following cell lysis. This mechanism might also explain the association that we did see with a gene-region SNP and a T-cell cytokine response. More work is needed to elucidate the complete role of HSP90B1 in the human immune response to infection.

## Supporting information

S1 Table Participant characteristics of South Africa pediatric tuberculosis genetic cohort study. CMA = Cape Mixed Ancestry.
(DOCX)

S2 Table Genotypic and allelic frequencies of SNPs included in this study. Results of the Pearson’s *x*^2^ test for the genotypic model are shown (*p*). Case = TB disease, Control = no TB disease. Chr12 Location = SNP location on chromosome 12 of the human genome using build GRCh38.p12.
(DOCX)

S3 Table Linkage disequilibrium R^2^ values for SNPs included within this study. Values were calculated using the control genotyping data from this cohort. This table can be compared to S7 Table, which provides linkage disequilibrium R^2^ values for the Yoruba in Ibadan population that was used to select haplotype-tagging SNPs for this study. Minor allele frequencies are shown at the convergence of the vertical and horizontal cells for each individual SNP (unshaded cells).
(DOCX)

S4 Table Genotype and allele frequencies from the Yoruba in Ibadan (YRI) population for SNPs that were included in our study. Data were generated from the 1000 Genomes Project phase 3 using Ensembl (www.ensembl.org). Note that for SNP rs11111854 and SNP rs2700509, the minor allele from the YRI population was the major allele in our study.
(DOCX)

S5 Table Linkage disequilibrium R^2^ values for relevant SNPs in the Yoruba in Ibadan (YRI) population. LD plot was generated using Ensembl (www.ensembl.org) for the YRI population from the 1000 Genomes Project phase 3. Minor allele frequencies are shown at the convergence of the vertical and horizontal cells for each individual SNP (unshaded cells). Note that for two SNPs (rs11111854 and rs2700509) the alternate allele was minor in our control population (S3 Table) relative to the YRI population.
(DOCX)

S6 Table Ancestry informative markers (AIMs) for cases and controls. Genotype and allele frequencies for ancestry informative markers (AIMs) are listed. Results of *x*^2^ testing for Hardy-Weinberg equilibrium are shown (HWE-p). No SNPs were in violation of HWE. Results of *x*^2^ testing using a genotypic model between cases and controls are also shown (Gen p). No significant differences between cases and controls were observed in any of the included SNPs.
(DOCX)

S7 Table Whole blood cytokine stimulation by *HSP90B1* SNP. General linearized model p-values are shown for interleukin 2 (IL-2) and interferon gamma (IFN-γ) stratified by SNP genotype.
(DOCX)

S8 Table Intracellular cytokine staining, by select *HSP90B1* SNP. General linearized model p-values are shown for CD4 positive (CD4) and CD8 positive (CD8) subsets, stratified by *HSP90B1* genotype.
(DOCX)

S9 Table Genetic association data stratified by ethnicity. Genotypic frequencies and results of the Pearson’s *x*^2^ test for the dominant model are shown for selected SNPs based on self-described ethnicity. For individuals who self-identified as Black and for whom more detailed ethnicity data were available, ~93% identified as Xhosa and ~6% as Sotho. CMA = Cape Mixed African. OR = odds ratio. 95% CI = 95% confidence interval.
(DOCX)

S10 Table Genetic association data for Cape Mixed Ancestry adjusted for AIMs. Cape Mixed Ancestry ethnicity genotype frequencies for select SNPs for individuals within for whom ancestry informative marker (AIMs) data were available. AIMs adjusted statistics are provided for cases versus controls under a dominant model. OR = odds ratio. 95% CI = 95% confidence interval.
(DOCX)

S11 Table Sensitivity analysis of TB genetic association. Data from sensitivity analysis includes only individuals with a more stringent case or control definition. For the sensitivity analysis, a TB case was defined as individuals with ‘Definite’ and ‘Probable’ TB diagnoses, with individuals carrying a ‘Possible TB’ diagnosis excluded. Controls were defined as individuals with ‘Not TB’; individuals carrying the ‘Unlikely TB’ diagnosis were excluded. Genotypic frequencies and results of the Pearson’s *x*^2^ test for the dominant model are shown for selected SNPs.
(DOCX)

S1 Fig rs1305392 is not associated with *HSP90B1* gene expression. Gene expression was compared to expression of GAPDH in unstimulated monocytes (A), dendritic cells (B), and T-cells (C) and stratified by genotype. Median and interquartile ranges are included and sample sizes are provided in parentheses below each genotype. Results were not significant for any cell type (*p*>0.05, ANOVA).
(DOCX)
