# Correction: Toll-like receptor chaperone *HSP90B1* and the immune response to *Mycobacteria*

**DOI:** 10.1371/journal.pone.0235935

**Published:** 2020-07-02

**Authors:** The PLOS ONE Staff

The seventh author's name is spelled incorrectly. The correct name is: Augustine Ajuogu. The publisher apologizes for the error.
