# Supplementary material for: Toll-like receptor chaperone HSP90B1 and the immune response to Mycobacteria
Source: PLoS One. 2018 Dec 14;13(12):e0208940. doi: 10.1371/journal.pone.0208940 (PMC6294361; doi:10.1371/journal.pone.0208940)
Supplement: S1 Table — CMA = Cape Mixed Ancestry. (DOCX) [file pone.0208940.s001.docx]

|  | Case N | Case (%) | Control N | Control (%) | Total |
| --- | --- | --- | --- | --- | --- |
| Total | 217 | 26.4 | 604 | 73.6 | 821 |
|  |  |  |  |  |  |
| Gender |  |  |  |  |  |
| Male | 112 | 51.6 | 280 | 46.5 | 392 |
| Female | 105 | 48.4 | 324 | 53.8 | 429 |
|  |  |  |  |  |  |
| Ethnicity |  |  |  |  |  |
| CMA | 197 | 90.8 | 482 | 80.1 | 679 |
| Black | 20 | 9.2 | 122 | 20.3 | 142 |
|  |  |  |  |  |  |
| TB Diagnosis |  |  |  |  |  |
| Not TB | 0 | 0 | 604 | 100.0 |  |
| Definite TB | 68 | 31.3 | 0 | 0 |  |
| Possible TB | 38 | 17.5 | 0 | 0 |  |
| Probable TB | 111 | 51.2 | 0 | 0 |  |
