# Supplementary material for: Toll-like receptor chaperone HSP90B1 and the immune response to Mycobacteria
Source: PLoS One. 2018 Dec 14;13(12):e0208940. doi: 10.1371/journal.pone.0208940 (PMC6294361; doi:10.1371/journal.pone.0208940)
Supplement: S6 Table — Genotype and allele frequencies for ancestry informative markers (AIMs) are listed. Results of x2 testing for Hardy-Weinberg equilibrium are shown (HWE-p). No SNPs were in violation of HWE. Results of x2 testing using a genotypic model between cases and controls are also shown (Gen p). No significant differences between cases and controls were observed in any of the included SNPs. (DOCX) [file pone.0208940.s006.docx]

|  |  |  | **Genotype** | | | | | | **Allele** | |  |  |
| --- | --- | --- | --- | --- | --- | --- | --- | --- | --- | --- | --- | --- |
| **AIMs SNP** | **Group** | **n** | **AA** | **Aa** | **aa** | **AA freq** | **Aa freq** | **aa freq** | **A freq** | **a freq** | **HWE p** | **Gen p** |
| rs10493578 | Control | 276 | 198 | 73 | 5 | 0.717 | 0.264 | 0.018 | 0.850 | 0.150 | 0.559 | 0.330 |
|  | Case | 133 | 88 | 40 | 5 | 0.662 | 0.301 | 0.038 | 0.812 | 0.188 |  |  |
| rs6008817 | Control | 278 | 133 | 114 | 31 | 0.478 | 0.410 | 0.112 | 0.683 | 0.317 | 0.383 | 0.803 |
|  | Case | 133 | 68 | 52 | 13 | 0.511 | 0.391 | 0.098 | 0.707 | 0.293 |  |  |
| rs137269 | Control | 276 | 107 | 129 | 40 | 0.388 | 0.467 | 0.145 | 0.621 | 0.379 | 0.912 | 0.515 |
|  | Case | 132 | 59 | 55 | 18 | 0.447 | 0.417 | 0.136 | 0.655 | 0.345 |  |  |
| rs7091054 | Control | 275 | 206 | 67 | 2 | 0.749 | 0.244 | 0.007 | 0.871 | 0.129 | 0.166 | 0.104 |
|  | Case | 133 | 104 | 25 | 4 | 0.782 | 0.188 | 0.030 | 0.876 | 0.124 |  |  |
| rs12026956 | Control | 276 | 77 | 135 | 64 | 0.279 | 0.489 | 0.232 | 0.524 | 0.476 | 0.745 | 0.815 |
|  | Case | 132 | 34 | 69 | 29 | 0.258 | 0.523 | 0.220 | 0.519 | 0.481 |  |  |
| rs16987743 | Control | 277 | 98 | 140 | 39 | 0.354 | 0.505 | 0.141 | 0.606 | 0.394 | 0.327 | 0.256 |
|  | Case | 133 | 57 | 56 | 20 | 0.429 | 0.421 | 0.150 | 0.639 | 0.361 |  |  |
| rs1572018 | Control | 275 | 158 | 99 | 18 | 0.575 | 0.360 | 0.065 | 0.755 | 0.245 | 0.641 | 0.445 |
|  | Case | 132 | 70 | 49 | 13 | 0.530 | 0.371 | 0.098 | 0.716 | 0.284 |  |  |
| rs12776665 | Control | 277 | 188 | 81 | 8 | 0.679 | 0.292 | 0.029 | 0.825 | 0.175 | 0.838 | 0.981 |
|  | Case | 133 | 89 | 40 | 4 | 0.669 | 0.301 | 0.030 | 0.820 | 0.180 |  |  |
| rs7165405 | Control | 276 | 74 | 152 | 50 | 0.268 | 0.551 | 0.181 | 0.543 | 0.457 | 0.068 | 0.640 |
|  | Case | 133 | 36 | 68 | 29 | 0.271 | 0.511 | 0.218 | 0.526 | 0.474 |  |  |
| rs2026914 | Control | 277 | 86 | 137 | 54 | 0.310 | 0.495 | 0.195 | 0.558 | 0.442 | 0.966 | 0.963 |
|  | Case | 133 | 43 | 65 | 25 | 0.323 | 0.489 | 0.188 | 0.568 | 0.432 |  |  |
| rs1544396 | Control | 276 | 117 | 119 | 40 | 0.424 | 0.431 | 0.145 | 0.639 | 0.361 | 0.281 | 0.459 |
|  | Case | 133 | 50 | 58 | 25 | 0.376 | 0.436 | 0.188 | 0.594 | 0.406 |  |  |
| rs1861760 | Control | 275 | 181 | 84 | 10 | 0.658 | 0.305 | 0.036 | 0.811 | 0.189 | 0.948 | 0.399 |
|  | Case | 133 | 96 | 34 | 3 | 0.722 | 0.256 | 0.023 | 0.850 | 0.150 |  |  |
| rs12132696 | Control | 275 | 140 | 110 | 25 | 0.509 | 0.400 | 0.091 | 0.709 | 0.291 | 0.614 | 0.363 |
|  | Case | 132 | 77 | 44 | 11 | 0.583 | 0.333 | 0.083 | 0.750 | 0.250 |  |  |
| rs1437787 | Control | 275 | 173 | 85 | 17 | 0.629 | 0.309 | 0.062 | 0.784 | 0.216 | 0.142 | 0.016 |
|  | Case | 132 | 65 | 60 | 7 | 0.492 | 0.455 | 0.053 | 0.720 | 0.280 |  |  |
| rs37268 | Control | 276 | 120 | 125 | 31 | 0.435 | 0.453 | 0.112 | 0.661 | 0.339 | 0.856 | 0.618 |
|  | Case | 133 | 61 | 54 | 18 | 0.459 | 0.406 | 0.135 | 0.662 | 0.338 |  |  |
| rs6445860 | Control | 278 | 134 | 117 | 27 | 0.482 | 0.421 | 0.097 | 0.692 | 0.308 | 0.843 | 0.599 |
|  | Case | 133 | 58 | 63 | 12 | 0.436 | 0.474 | 0.090 | 0.673 | 0.327 |  |  |
| rs1942885 | Control | 275 | 85 | 129 | 61 | 0.309 | 0.469 | 0.222 | 0.544 | 0.456 | 0.365 | 0.356 |
|  | Case | 133 | 34 | 62 | 37 | 0.256 | 0.466 | 0.278 | 0.489 | 0.511 |  |  |
| rs10898250 | Control | 277 | 99 | 147 | 31 | 0.357 | 0.531 | 0.112 | 0.623 | 0.377 | 0.031 | 0.001 |
|  | Case | 132 | 32 | 68 | 32 | 0.242 | 0.515 | 0.242 | 0.500 | 0.500 |  |  |
| rs1348587 | Control | 276 | 116 | 127 | 33 | 0.420 | 0.460 | 0.120 | 0.650 | 0.350 | 0.845 | 0.736 |
|  | Case | 133 | 61 | 56 | 16 | 0.459 | 0.421 | 0.120 | 0.669 | 0.331 |  |  |
| rs2579785 | Control | 278 | 73 | 135 | 70 | 0.263 | 0.486 | 0.252 | 0.505 | 0.495 | 0.633 | 0.482 |
|  | Case | 133 | 30 | 73 | 30 | 0.226 | 0.549 | 0.226 | 0.500 | 0.500 |  |  |
| rs2554832 | Control | 276 | 107 | 127 | 42 | 0.388 | 0.460 | 0.152 | 0.618 | 0.382 | 0.670 | 0.728 |
|  | Case | 133 | 56 | 60 | 17 | 0.421 | 0.451 | 0.128 | 0.647 | 0.353 |  |  |
| rs1800007 | Control | 277 | 143 | 115 | 19 | 0.516 | 0.415 | 0.069 | 0.724 | 0.276 | 0.523 | 0.330 |
|  | Case | 133 | 71 | 48 | 14 | 0.534 | 0.361 | 0.105 | 0.714 | 0.286 |  |  |
| rs1888207 | Control | 276 | 124 | 127 | 25 | 0.449 | 0.460 | 0.091 | 0.679 | 0.321 | 0.351 | 0.621 |
|  | Case | 133 | 56 | 61 | 16 | 0.421 | 0.459 | 0.120 | 0.650 | 0.350 |  |  |
| rs8117683 | Control | 277 | 192 | 80 | 5 | 0.693 | 0.289 | 0.018 | 0.838 | 0.162 | 0.308 | 0.470 |
|  | Case | 133 | 92 | 36 | 5 | 0.692 | 0.271 | 0.038 | 0.827 | 0.173 |  |  |
| rs12308701 | Control | 276 | 92 | 133 | 51 | 0.333 | 0.482 | 0.185 | 0.574 | 0.426 | 0.810 | 0.074 |
|  | Case | 133 | 57 | 61 | 15 | 0.429 | 0.459 | 0.113 | 0.658 | 0.342 |  |  |
| rs1486548 | Control | 275 | 128 | 110 | 37 | 0.465 | 0.400 | 0.135 | 0.665 | 0.335 | 0.092 | 0.055 |
|  | Case | 133 | 49 | 70 | 14 | 0.368 | 0.526 | 0.105 | 0.632 | 0.368 |  |  |
| rs11903376 | Control | 274 | 85 | 133 | 56 | 0.310 | 0.485 | 0.204 | 0.553 | 0.447 | 0.763 | 0.187 |
|  | Case | 133 | 30 | 70 | 33 | 0.226 | 0.526 | 0.248 | 0.489 | 0.511 |  |  |
| rs1470023 | Control | 274 | 104 | 135 | 35 | 0.380 | 0.493 | 0.128 | 0.626 | 0.374 | 0.388 | 0.934 |
|  | Case | 133 | 52 | 63 | 18 | 0.391 | 0.474 | 0.135 | 0.628 | 0.372 |  |  |
| rs1257017 | Control | 276 | 141 | 121 | 14 | 0.511 | 0.438 | 0.051 | 0.730 | 0.270 | 0.062 | 0.698 |
|  | Case | 133 | 70 | 54 | 9 | 0.526 | 0.406 | 0.068 | 0.729 | 0.271 |  |  |
| rs9311835 | Control | 274 | 168 | 92 | 14 | 0.613 | 0.336 | 0.051 | 0.781 | 0.219 | 0.761 | 0.545 |
|  | Case | 133 | 89 | 38 | 6 | 0.669 | 0.286 | 0.045 | 0.812 | 0.188 |  |  |
| rs1468920 | Control | 275 | 113 | 121 | 41 | 0.411 | 0.440 | 0.149 | 0.631 | 0.369 | 0.360 | 0.742 |
|  | Case | 132 | 57 | 59 | 16 | 0.432 | 0.447 | 0.121 | 0.655 | 0.345 |  |  |
| rs627238 | Control | 277 | 161 | 99 | 17 | 0.581 | 0.357 | 0.061 | 0.760 | 0.240 | 0.733 | 0.502 |
|  | Case | 133 | 85 | 42 | 6 | 0.639 | 0.316 | 0.045 | 0.797 | 0.203 |  |  |
| rs678701 | Control | 277 | 127 | 124 | 26 | 0.458 | 0.448 | 0.094 | 0.682 | 0.318 | 0.588 | 0.926 |
|  | Case | 132 | 60 | 61 | 11 | 0.455 | 0.462 | 0.083 | 0.686 | 0.314 |  |  |
| rs8068946 | Control | 275 | 146 | 107 | 22 | 0.531 | 0.389 | 0.080 | 0.725 | 0.275 | 0.700 | 0.905 |
|  | Case | 132 | 72 | 51 | 9 | 0.545 | 0.386 | 0.068 | 0.739 | 0.261 |  |  |
| rs10853034 | Control | 276 | 68 | 137 | 71 | 0.246 | 0.496 | 0.257 | 0.495 | 0.505 | 0.906 | 0.408 |
|  | Case | 132 | 31 | 74 | 27 | 0.235 | 0.561 | 0.205 | 0.515 | 0.485 |  |  |
| rs4841295 | Control | 277 | 159 | 105 | 13 | 0.574 | 0.379 | 0.047 | 0.764 | 0.236 | 0.408 | 0.193 |
|  | Case | 133 | 69 | 52 | 12 | 0.519 | 0.391 | 0.090 | 0.714 | 0.286 |  |  |
| rs7584977 | Control | 276 | 98 | 128 | 50 | 0.355 | 0.464 | 0.181 | 0.587 | 0.413 | 0.470 | 0.723 |
|  | Case | 132 | 42 | 63 | 27 | 0.318 | 0.477 | 0.205 | 0.557 | 0.443 |  |  |
| rs596985 | Control | 277 | 85 | 128 | 64 | 0.307 | 0.462 | 0.231 | 0.538 | 0.462 | 0.241 | 0.171 |
|  | Case | 132 | 39 | 72 | 21 | 0.295 | 0.545 | 0.159 | 0.568 | 0.432 |  |  |
| rs16956174 | Control | 275 | 72 | 144 | 59 | 0.262 | 0.524 | 0.215 | 0.524 | 0.476 | 0.411 | 0.520 |
|  | Case | 133 | 42 | 64 | 27 | 0.316 | 0.481 | 0.203 | 0.556 | 0.444 |  |  |
| rs6598891 | Control | 277 | 69 | 138 | 70 | 0.249 | 0.498 | 0.253 | 0.498 | 0.502 | 0.952 | 0.513 |
|  | Case | 133 | 39 | 66 | 28 | 0.293 | 0.496 | 0.211 | 0.541 | 0.459 |  |  |
| rs10519410 | Control | 278 | 92 | 135 | 51 | 0.331 | 0.486 | 0.183 | 0.574 | 0.426 | 0.905 | 0.464 |
|  | Case | 133 | 36 | 71 | 26 | 0.271 | 0.534 | 0.195 | 0.538 | 0.462 |  |  |
| rs10127540 | Control | 275 | 146 | 106 | 23 | 0.531 | 0.385 | 0.084 | 0.724 | 0.276 | 0.547 | 0.871 |
|  | Case | 133 | 68 | 52 | 13 | 0.511 | 0.391 | 0.098 | 0.707 | 0.293 |  |  |
| rs4909722 | Control | 276 | 97 | 144 | 35 | 0.351 | 0.522 | 0.127 | 0.612 | 0.388 | 0.100 | 0.453 |
|  | Case | 133 | 49 | 62 | 22 | 0.368 | 0.466 | 0.165 | 0.602 | 0.398 |  |  |
| rs6927607 | Control | 276 | 119 | 124 | 33 | 0.431 | 0.449 | 0.120 | 0.656 | 0.344 | 0.936 | 0.819 |
|  | Case | 132 | 58 | 61 | 13 | 0.439 | 0.462 | 0.098 | 0.670 | 0.330 |  |  |
| rs2710994 | Control | 277 | 74 | 151 | 52 | 0.267 | 0.545 | 0.188 | 0.540 | 0.460 | 0.106 | 0.733 |
|  | Case | 133 | 39 | 67 | 27 | 0.293 | 0.504 | 0.203 | 0.545 | 0.455 |  |  |
| rs7852195 | Control | 275 | 75 | 129 | 71 | 0.273 | 0.469 | 0.258 | 0.507 | 0.493 | 0.307 | 0.788 |
|  | Case | 133 | 40 | 62 | 31 | 0.301 | 0.466 | 0.233 | 0.534 | 0.466 |  |  |
| rs9881149 | Control | 277 | 76 | 130 | 71 | 0.274 | 0.469 | 0.256 | 0.509 | 0.491 | 0.309 | 0.517 |
|  | Case | 133 | 42 | 63 | 28 | 0.316 | 0.474 | 0.211 | 0.553 | 0.447 |  |  |
| rs6747116 | Control | 277 | 175 | 94 | 8 | 0.632 | 0.339 | 0.029 | 0.801 | 0.199 | 0.270 | 0.223 |
|  | Case | 133 | 94 | 34 | 5 | 0.707 | 0.256 | 0.038 | 0.835 | 0.165 |  |  |
| rs7657948 | Control | 277 | 109 | 118 | 50 | 0.394 | 0.426 | 0.181 | 0.606 | 0.394 | 0.074 | 0.408 |
|  | Case | 132 | 50 | 64 | 18 | 0.379 | 0.485 | 0.136 | 0.621 | 0.379 |  |  |
| rs2901235 | Control | 275 | 121 | 122 | 32 | 0.440 | 0.444 | 0.116 | 0.662 | 0.338 | 0.882 | 0.788 |
|  | Case | 133 | 60 | 55 | 18 | 0.451 | 0.414 | 0.135 | 0.658 | 0.342 |  |  |
| rs1941411 | Control | 277 | 173 | 88 | 16 | 0.625 | 0.318 | 0.058 | 0.783 | 0.217 | 0.288 | 0.903 |
|  | Case | 133 | 80 | 45 | 8 | 0.602 | 0.338 | 0.060 | 0.771 | 0.229 |  |  |
| rs2101523 | Control | 276 | 101 | 135 | 40 | 0.366 | 0.489 | 0.145 | 0.611 | 0.389 | 0.636 | 0.320 |
|  | Case | 133 | 47 | 59 | 27 | 0.353 | 0.444 | 0.203 | 0.575 | 0.425 |  |  |
| rs6719593 | Control | 276 | 110 | 109 | 57 | 0.399 | 0.395 | 0.207 | 0.596 | 0.404 | 0.003 | 0.027 |
|  | Case | 133 | 37 | 70 | 26 | 0.278 | 0.526 | 0.195 | 0.541 | 0.459 |  |  |
| rs2249698 | Control | 278 | 102 | 131 | 45 | 0.367 | 0.471 | 0.162 | 0.603 | 0.397 | 0.787 | 0.535 |
|  | Case | 132 | 41 | 68 | 23 | 0.311 | 0.515 | 0.174 | 0.568 | 0.432 |  |  |
| rs17469306 | Control | 275 | 180 | 79 | 16 | 0.655 | 0.287 | 0.058 | 0.798 | 0.202 | 0.072 | 0.808 |
|  | Case | 133 | 86 | 41 | 6 | 0.647 | 0.308 | 0.045 | 0.801 | 0.199 |  |  |
| rs802372 | Control | 276 | 101 | 128 | 47 | 0.366 | 0.464 | 0.170 | 0.598 | 0.402 | 0.555 | 0.397 |
|  | Case | 133 | 44 | 59 | 30 | 0.331 | 0.444 | 0.226 | 0.553 | 0.447 |  |  |
| rs2919308 | Control | 277 | 141 | 123 | 13 | 0.509 | 0.444 | 0.047 | 0.731 | 0.269 | 0.032 | 0.070 |
|  | Case | 133 | 79 | 44 | 10 | 0.594 | 0.331 | 0.075 | 0.759 | 0.241 |  |  |
| rs13300278 | Control | 277 | 107 | 134 | 36 | 0.386 | 0.484 | 0.130 | 0.628 | 0.372 | 0.554 | 0.481 |
|  | Case | 133 | 51 | 59 | 23 | 0.383 | 0.444 | 0.173 | 0.605 | 0.395 |  |  |
| rs10194455 | Control | 275 | 84 | 144 | 47 | 0.305 | 0.524 | 0.171 | 0.567 | 0.433 | 0.270 | 0.172 |
|  | Case | 133 | 39 | 61 | 33 | 0.293 | 0.459 | 0.248 | 0.523 | 0.477 |  |  |
| rs8057341 | Control | 277 | 135 | 116 | 26 | 0.487 | 0.419 | 0.094 | 0.697 | 0.303 | 0.881 | 0.069 |
|  | Case | 133 | 59 | 51 | 23 | 0.444 | 0.383 | 0.173 | 0.635 | 0.365 |  |  |
| rs2836746 | Control | 275 | 138 | 113 | 24 | 0.502 | 0.411 | 0.087 | 0.707 | 0.293 | 0.899 | 0.450 |
|  | Case | 133 | 58 | 61 | 14 | 0.436 | 0.459 | 0.105 | 0.665 | 0.335 |  |  |
| rs2294654 | Control | 278 | 133 | 127 | 18 | 0.478 | 0.457 | 0.065 | 0.707 | 0.293 | 0.088 | 0.072 |
|  | Case | 133 | 50 | 68 | 15 | 0.376 | 0.511 | 0.113 | 0.632 | 0.368 |  |  |
| rs6685064 | Control | 275 | 98 | 132 | 45 | 0.356 | 0.480 | 0.164 | 0.596 | 0.404 | 0.961 | 0.792 |
|  | Case | 132 | 47 | 60 | 25 | 0.356 | 0.455 | 0.189 | 0.583 | 0.417 |  |  |
| rs700085 | Control | 277 | 83 | 138 | 56 | 0.300 | 0.498 | 0.202 | 0.549 | 0.451 | 0.921 | 0.304 |
|  | Case | 133 | 31 | 69 | 33 | 0.233 | 0.519 | 0.248 | 0.492 | 0.508 |  |  |
| rs1744849 | Control | 276 | 170 | 91 | 15 | 0.616 | 0.330 | 0.054 | 0.781 | 0.219 | 0.541 | 0.544 |
|  | Case | 133 | 85 | 44 | 4 | 0.639 | 0.331 | 0.030 | 0.805 | 0.195 |  |  |
| rs2823877 | Control | 274 | 88 | 136 | 50 | 0.321 | 0.496 | 0.182 | 0.569 | 0.431 | 0.840 | 0.904 |
|  | Case | 133 | 44 | 63 | 26 | 0.331 | 0.474 | 0.195 | 0.568 | 0.432 |  |  |
| rs10856819 | Control | 276 | 73 | 137 | 66 | 0.264 | 0.496 | 0.239 | 0.513 | 0.487 | 0.913 | 0.183 |
|  | Case | 133 | 43 | 68 | 22 | 0.323 | 0.511 | 0.165 | 0.579 | 0.421 |  |  |
| rs16913918 | Control | 276 | 81 | 146 | 49 | 0.293 | 0.529 | 0.178 | 0.558 | 0.442 | 0.229 | 0.585 |
|  | Case | 132 | 38 | 65 | 29 | 0.288 | 0.492 | 0.220 | 0.534 | 0.466 |  |  |
| rs1024196 | Control | 278 | 88 | 123 | 67 | 0.317 | 0.442 | 0.241 | 0.538 | 0.462 | 0.067 | 0.682 |
|  | Case | 133 | 37 | 60 | 36 | 0.278 | 0.451 | 0.271 | 0.504 | 0.496 |  |  |
| rs1426955 | Control | 276 | 96 | 133 | 47 | 0.348 | 0.482 | 0.170 | 0.589 | 0.411 | 0.936 | 0.060 |
|  | Case | 132 | 36 | 61 | 35 | 0.273 | 0.462 | 0.265 | 0.504 | 0.496 |  |  |
| rs7671283 | Control | 277 | 132 | 116 | 29 | 0.477 | 0.419 | 0.105 | 0.686 | 0.314 | 0.640 | 0.313 |
|  | Case | 133 | 54 | 60 | 19 | 0.406 | 0.451 | 0.143 | 0.632 | 0.368 |  |  |
| rs4904690 | Control | 277 | 196 | 71 | 10 | 0.708 | 0.256 | 0.036 | 0.836 | 0.164 | 0.269 | 0.942 |
|  | Case | 133 | 96 | 32 | 5 | 0.722 | 0.241 | 0.038 | 0.842 | 0.158 |  |  |
| rs8056754 | Control | 276 | 173 | 89 | 14 | 0.627 | 0.322 | 0.051 | 0.788 | 0.212 | 0.564 | 0.387 |
|  | Case | 133 | 88 | 42 | 3 | 0.662 | 0.316 | 0.023 | 0.820 | 0.180 |  |  |
| rs2019340 | Control | 276 | 100 | 139 | 37 | 0.362 | 0.504 | 0.134 | 0.614 | 0.386 | 0.298 | 0.465 |
|  | Case | 133 | 45 | 64 | 24 | 0.338 | 0.481 | 0.180 | 0.579 | 0.421 |  |  |
| rs1337775 | Control | 275 | 109 | 122 | 44 | 0.396 | 0.444 | 0.160 | 0.618 | 0.382 | 0.318 | 0.335 |
|  | Case | 133 | 62 | 55 | 16 | 0.466 | 0.414 | 0.120 | 0.673 | 0.327 |  |  |
| rs4897445 | Control | 276 | 169 | 98 | 9 | 0.612 | 0.355 | 0.033 | 0.790 | 0.210 | 0.248 | 0.492 |
|  | Case | 133 | 87 | 40 | 6 | 0.654 | 0.301 | 0.045 | 0.805 | 0.195 |  |  |
| rs11197672 | Control | 275 | 113 | 123 | 39 | 0.411 | 0.447 | 0.142 | 0.635 | 0.365 | 0.555 | 0.976 |
|  | Case | 133 | 56 | 58 | 19 | 0.421 | 0.436 | 0.143 | 0.639 | 0.361 |  |  |
| rs16838138 | Control | 275 | 121 | 123 | 31 | 0.440 | 0.447 | 0.113 | 0.664 | 0.336 | 0.976 | 0.795 |
|  | Case | 133 | 63 | 55 | 15 | 0.474 | 0.414 | 0.113 | 0.680 | 0.320 |  |  |
| rs1003395 | Control | 277 | 120 | 122 | 35 | 0.433 | 0.440 | 0.126 | 0.653 | 0.347 | 0.646 | 0.996 |
|  | Case | 133 | 57 | 59 | 17 | 0.429 | 0.444 | 0.128 | 0.650 | 0.350 |  |  |
| rs4281695 | Control | 275 | 102 | 118 | 55 | 0.371 | 0.429 | 0.200 | 0.585 | 0.415 | 0.054 | 0.439 |
|  | Case | 133 | 43 | 66 | 24 | 0.323 | 0.496 | 0.180 | 0.571 | 0.429 |  |  |
| rs2849266 | Control | 276 | 114 | 121 | 41 | 0.413 | 0.438 | 0.149 | 0.632 | 0.368 | 0.342 | 0.324 |
|  | Case | 133 | 55 | 65 | 13 | 0.414 | 0.489 | 0.098 | 0.658 | 0.342 |  |  |
| rs820335 | Control | 276 | 72 | 145 | 59 | 0.261 | 0.525 | 0.214 | 0.524 | 0.476 | 0.378 | 0.933 |
|  | Case | 133 | 37 | 68 | 28 | 0.278 | 0.511 | 0.211 | 0.534 | 0.466 |  |  |
| rs4233905 | Control | 276 | 78 | 138 | 60 | 0.283 | 0.500 | 0.217 | 0.533 | 0.467 | 0.943 | 0.559 |
|  | Case | 133 | 34 | 74 | 25 | 0.256 | 0.556 | 0.188 | 0.534 | 0.466 |  |  |
| rs7601 | Control | 274 | 103 | 126 | 45 | 0.376 | 0.460 | 0.164 | 0.606 | 0.394 | 0.539 | 0.124 |
|  | Case | 132 | 57 | 63 | 12 | 0.432 | 0.477 | 0.091 | 0.670 | 0.330 |  |  |
| rs2421069 | Control | 277 | 76 | 124 | 77 | 0.274 | 0.448 | 0.278 | 0.498 | 0.502 | 0.081 | 0.807 |
|  | Case | 133 | 35 | 64 | 34 | 0.263 | 0.481 | 0.256 | 0.504 | 0.496 |  |  |
| rs10957157 | Control | 276 | 180 | 84 | 12 | 0.652 | 0.304 | 0.043 | 0.804 | 0.196 | 0.583 | 0.348 |
|  | Case | 133 | 95 | 35 | 3 | 0.714 | 0.263 | 0.023 | 0.846 | 0.154 |  |  |
| rs1517835 | Control | 276 | 100 | 123 | 53 | 0.362 | 0.446 | 0.192 | 0.585 | 0.415 | 0.173 | 0.588 |
|  | Case | 132 | 43 | 66 | 23 | 0.326 | 0.500 | 0.174 | 0.576 | 0.424 |  |  |
| rs2251244 | Control | 277 | 149 | 106 | 22 | 0.538 | 0.383 | 0.079 | 0.729 | 0.271 | 0.606 | 0.483 |
|  | Case | 133 | 75 | 44 | 14 | 0.564 | 0.331 | 0.105 | 0.729 | 0.271 |  |  |
| rs1466020 | Control | 276 | 168 | 100 | 8 | 0.609 | 0.362 | 0.029 | 0.790 | 0.210 | 0.129 | 0.058 |
|  | Case | 133 | 84 | 39 | 10 | 0.632 | 0.293 | 0.075 | 0.778 | 0.222 |  |  |
| rs2549159 | Control | 275 | 144 | 105 | 26 | 0.524 | 0.382 | 0.095 | 0.715 | 0.285 | 0.288 | 0.605 |
|  | Case | 133 | 66 | 57 | 10 | 0.496 | 0.429 | 0.075 | 0.711 | 0.289 |  |  |
| rs10496882 | Control | 273 | 67 | 139 | 67 | 0.245 | 0.509 | 0.245 | 0.500 | 0.500 | 0.762 | 0.369 |
|  | Case | 133 | 39 | 58 | 36 | 0.293 | 0.436 | 0.271 | 0.511 | 0.489 |  |  |
| rs1113480 | Control | 276 | 145 | 102 | 29 | 0.525 | 0.370 | 0.105 | 0.710 | 0.290 | 0.089 | 0.253 |
|  | Case | 133 | 59 | 55 | 19 | 0.444 | 0.414 | 0.143 | 0.650 | 0.350 |  |  |
| rs1979537 | Control | 276 | 148 | 116 | 12 | 0.536 | 0.420 | 0.043 | 0.746 | 0.254 | 0.067 | 0.736 |
|  | Case | 132 | 68 | 56 | 8 | 0.515 | 0.424 | 0.061 | 0.727 | 0.273 |  |  |
