# Supplementary material for: Toll-like receptor chaperone HSP90B1 and the immune response to Mycobacteria
Source: PLoS One. 2018 Dec 14;13(12):e0208940. doi: 10.1371/journal.pone.0208940 (PMC6294361; doi:10.1371/journal.pone.0208940)
Supplement: S7 Table — General linearized model p-values are shown for interleukin 2 (IL-2) and interferon gamma (IFN-γ) stratified by SNP genotype. (DOCX) [file pone.0208940.s007.docx]

| **SNP** | **IL-2 (*p)*** | **IFN-γ** **(*p)*** |
| --- | --- | --- |
| rs2070908 | 0.939 | 0.502 |
| rs57781511 | 0.388 | 0.424 |
| rs1165681 | 0.443 | 0.73 |
| rs1920413 | 0.08 | 0.403 |
| rs3794240 | 0.888 | 0.454 |
| rs17034943 | 0.261 | 0.723 |
| rs1437502 | 0.14 | 0.75 |
| rs1305392 | 0.055 | 0.128 |
| rs1165687 | 0.767 | 0.742 |
| rs4964142 | 0.602 | 0.719 |
| rs2249210 | 0.499 | 0.486 |
| rs10507172 | 0.002 | 0.14 |
| rs7136550 | 0.141 | 0.146 |
| rs10507173 | 0.002 | 0.168 |
| rs11111854 | 0.194 | 0.429 |
| rs79579452 | 0.1 | 0.368 |
| rs2700509 | 0.307 | 0.969 |
