# Supplementary material for: Toll-like receptor chaperone HSP90B1 and the immune response to Mycobacteria
Source: PLoS One. 2018 Dec 14;13(12):e0208940. doi: 10.1371/journal.pone.0208940 (PMC6294361; doi:10.1371/journal.pone.0208940)
Supplement: S8 Table — General linearized model p-values are shown for CD4 positive (CD4) and CD8 positive (CD8) subsets, stratified by HSP90B1 genotype. (DOCX) [file pone.0208940.s008.docx]

| **SNP** | **CD4 IL2 (*p*)** | **CD4 IFN-γ (*p*)** | **CD8 IL-2 (*p)*** | **CD8 IFN-γ (*p*)** |
| --- | --- | --- | --- | --- |
| rs1920413 | 0.71 | 0.31 | 0.33 | 0.74 |
| rs1305392 | 0.4 | 0.17 | 0.63 | 0.27 |
| rs10507172 | 0.3 | 0.66 | 0.58 | 0.88 |
| rs10507173 | <0.0001 | 0.6 | 0.6 | 0.8 |
