# Supplementary material for: Toll-like receptor chaperone HSP90B1 and the immune response to Mycobacteria
Source: PLoS One. 2018 Dec 14;13(12):e0208940. doi: 10.1371/journal.pone.0208940 (PMC6294361; doi:10.1371/journal.pone.0208940)
Supplement: Supplementary file 5 [file pone.0208940.s002.docx]

| | | | Genotype | | | | | | Alleles | | | | *p* |
|---|---|---|---|---|---|---|---|---|---|---|---|---|---|
| SNP | Chr12 Location | Group | Major (N) | Het (N) | Minor (N) | Major (%) | Het (%) | Minor (%) | Major (N) | Minor (N) | Major (%) | Minor (%) | |
| rs2070908 | 103,930,404 | | C/C | C/G | G/G | C/C | C/G | G/G | C | G | C | G | |
| | | Control | 230 | 277 | 92 | 38.4 | 46.2 | 15.4 | 737 | 461 | 61.5 | 38.5 | |
| | | Case | 74 | 102 | 37 | 34.7 | 47.9 | 17.4 | 250 | 176 | 58.7 | 41.3 | 0.59 |
| rs57781511 | 103,930,667 | | G/G | G/A | A/A | G/G | G/A | A/A | G | A | G | A | |
| | | Control | 432 | 154 | 13 | 72.1 | 25.7 | 2.2 | 1018 | 180 | 85.0 | 15.0 | |
| | | Case | 149 | 62 | 3 | 69.6 | 29.0 | 1.4 | 360 | 68 | 84.1 | 15.9 | 0.54 |
| rs1165681 | 103,931,333 | | C/C | C/T | T/T | C/C | C/T | T/T | C | T | C | T | |
| | | Control | 159 | 321 | 121 | 26.5 | 53.4 | 20.1 | 639 | 563 | 53.2 | 46.8 | |
| | | Case | 68 | 106 | 38 | 32.1 | 50.0 | 17.9 | 242 | 182 | 57.1 | 42.9 | 0.29 |
| rs1920413 | 103,933,213 | | G/G | G/A | A/A | G/G | G/A | A/A | G | A | G | A | |
| | | Control | 222 | 306 | 70 | 37.1 | 51.2 | 11.7 | 750 | 446 | 62.7 | 37.3 | |
| | | Case | 95 | 95 | 25 | 44.2 | 44.2 | 11.6 | 285 | 145 | 66.3 | 33.7 | 0.17 |
| rs3794240 | 103,935,763 | | G/G | G/T | T/T | G/G | G/T | T/T | G | T | G | T | |
| | | Control | 347 | 210 | 47 | 57.5 | 34.8 | 7.8 | 904 | 304 | 74.8 | 25.2 | |
| | | Case | 125 | 75 | 17 | 57.6 | 34.6 | 7.8 | 325 | 109 | 74.9 | 25.1 | 1 |
| rs17034943 | 103,936,172 | | G/G | G/A | A/A | G/G | G/A | A/A | G | A | G | A | |
| | | Control | 431 | 156 | 16 | 71.5 | 25.9 | 2.7 | 1018 | 188 | 84.4 | 15.6 | |
| | | Case | 150 | 58 | 8 | 69.4 | 26.9 | 3.7 | 358 | 74 | 82.9 | 17.1 | 0.69 |
| rs1437502 | 103,936,604 | | G/G | G/A | A/A | G/G | G/A | A/A | G | A | G | A | |
| | | Control | 104 | 91 | 17 | 49.1 | 42.9 | 8.0 | 299 | 125 | 70.5 | 29.5 | |
| | | Case | 272 | 280 | 44 | 45.6 | 47.0 | 7.4 | 824 | 368 | 69.1 | 30.9 | 0.6 |
| rs1305392 | 103,939,267 | | T/T | T/G | G/G | T/T | T/G | G/G | T | G | T | G | |
| | | Control | 227 | 271 | 105 | 37.6 | 44.9 | 17.4 | 725 | 481 | 60.1 | 39.9 | |
| | | Case | 71 | 103 | 43 | 32.7 | 47.5 | 19.8 | 245 | 189 | 56.5 | 43.5 | 0.41 |
| rs1165687 | 103,940,415 | | C/C | C/A | A/A | C/C | C/A | A/A | C | A | C | A | |
| | | Control | 381 | 184 | 36 | 63.4 | 30.6 | 6 | 946 | 256 | 78.7 | 21.3 | |
| | | Case | 139 | 67 | 9 | 64.7 | 31.2 | 4.2 | 345 | 85 | 80.2 | 19.8 | 0.61 |
| rs4964142 | 103,946,106 | | C/C | C/G | G/G | C/C | C/G | G/G | C | G | C | G | |
| | | Control | 498 | 95 | 7 | 83.0 | 15.8 | 1.2 | 1091 | 109 | 90.9 | 9.1 | |
| | | Case | 174 | 39 | 2 | 80.9 | 18.1 | 0.9 | 387 | 43 | 90.0 | 10.0 | 0.71 |
| rs2249210 | 103,948,534 | | T/T | T/C | C/C | | | | | | | | |
| | | Control | 373 | 197 | 30 | 62.2 | 32.8 | 5.0 | 943 | 257 | 78.6 | 21.4 | |
| | | Case | 139 | 69 | 7 | 64.7 | 32.1 | 3.3 | 347 | 83 | 80.7 | 19.3 | 0.54 |
| rs10507172 | 103,949,490 | | G/G | G/A | A/A | G/G | G/A | A/A | G | A | G | A | |
| | | Control | 406 | 179 | 17 | 67.4 | 29.7 | 2.8 | 991 | 213 | 82.3 | 17.7 | |
| | | Case | 163 | 49 | 5 | 75.1 | 22.6 | 2.3 | 375 | 59 | 86.4 | 13.6 | 0.11 |
| rs7136550 | 103,949,737 | | G/G | G/C | C/C | G/G | G/C | C/C | G | C | G | C | |
| | | Control | 347 | 210 | 44 | 57.7 | 34.9 | 7.3 | 904 | 298 | 75.2 | 24.8 | |
| | | Case | 116 | 89 | 9 | 54.2 | 41.6 | 4.2 | 321 | 107 | 75.0 | 25.0 | 0.1 |
| rs10507173 | 103,950,058 | | A/A | G/A | G/G | A/A | G/A | G/G | A | G | A | G | |
| | | Control | 467 | 128 | 7 | 77.6 | 21.3 | 1.2 | 1062 | 142 | 88.2 | 11.8 | |
| | | Case | 184 | 31 | 2 | 84.8 | 14.3 | 0.9 | 399 | 35 | 91.9 | 8.1 | 0.077 |
| rs11111854 | 103,951,475 | | A/A | G/A | G/G | A/A | G/A | G/G | A | G | A | G | |
| | | Control | 257 | 277 | 67 | 42.8 | 46.1 | 11.1 | 791 | 411 | 65.8 | 34.2 | |
| | | Case | 98 | 91 | 27 | 45.4 | 42.1 | 12.5 | 287 | 145 | 66.4 | 33.6 | 0.59 |
| rs79579452 | 103,952,170 | | T/T | T/C | C/C | T/T | T/C | C/C | T | C | T | C | |
| | | Control | 558 | 19 | 0 | 96.7 | 3.3 | 0.0 | 1135 | 19 | 98.4 | 1.6 | |
| | | Case | 198 | 46 | 0 | 81.1 | 18.9 | 0.0 | 442 | 46 | 90.6 | 9.4 | 0.59 |
| rs2700509 | 103,952,641 | | G/G | G/A | A/A | G/G | G/A | A/A | G | A | G | A | |
| | | Control | 183 | 275 | 142 | 30.5 | 45.8 | 23.7 | 641 | 559 | 53.4 | 46.6 | |
| | | Case | 72 | 102 | 41 | 33.5 | 47.4 | 19.1 | 246 | 184 | 57.2 | 42.8 | 0.36 |
