# Supplementary material for: Toll-like receptor chaperone HSP90B1 and the immune response to Mycobacteria
Source: PLoS One. 2018 Dec 14;13(12):e0208940. doi: 10.1371/journal.pone.0208940 (PMC6294361; doi:10.1371/journal.pone.0208940)
Supplement: Supplementary file 7 [file pone.0208940.s004.docx]

		Genotype						Alleles			
SNP	Chr12 Location	Major (N)	Het (N)	Minor (N)	Major (%)	Het (%)	Minor (%)	Major (N)	Minor (N)	Major (%)	Minor (%)
rs2070908	103,930,404	C/C	C/G	G/G	C/C	C/G	G/G	C	G	C	G
		32	57	19	29.6	52.8	17.6	121	95	56.0	44.0
rs57781511	103,930,667	G/G	G/A	A/A	G/G	G/A	A/A	G	A	G	A
		70	35	3	64.8	32.4	2.8	175	41	81.0	19.0
rs1165681	103,931,333	C/C	C/T	T/T	C/C	C/T	T/T	C	T	C	T
		38	55	15	35.2	50.9	13.9	131	85	60.6	39.4
rs1920413	103,933,213	G/G	G/A	A/A	G/G	G/A	A/A	G	A	G	A
		54	40	14	50.0	37.0	13.0	148	68	68.5	31.5
rs17034938	103,935,434	A/A	A/G	G/G	A/A	A/G	G/G	A	G	A	G
		65	35	8	60.2	32.4	7.4	165	51	76.4	23.6
rs3794240	103,935,763	G/G	G/T	T/T	G/G	G/T	T/T	G	T	G	T
		67	38	3	62.0	35.2	2.8	172	44	79.6	20.4
rs17034943	103,936,172	G/G	G/A	A/A	G/G	G/A	A/A	G	A	G	A
		79	25	4	73.1	23.1	3.7	183	33	84.7	15.3
rs1437502	103,936,604	G/G	G/A	A/A	G/G	G/A	A/A	G	A	G	A
		36	53	19	33.3	49.1	17.6	125	91	57.9	42.1
rs1305392	103,939,267	T/T	T/G	G/G	T/T	T/G	G/G	T	G	T	G
		59	42	7	54.6	38.9	6.5	160	56	74.1	25.9
rs1165687	103,940,415	C/C	C/A	A/A	C/C	C/A	A/A	C	A	C	A
		63	38	7	58.3	35.2	6.5	164	52	75.9	24.1
rs4964142	103,946,106	C/C	C/G	G/G	C/C	C/G	G/G	C	G	C	G
		80	22	6	74.1	20.4	5.6	182	34	84.3	15.7
rs7313381	103,948,026	C/C	C/T	T/T	C/C	C/T	T/T	C	T	C	T
		78	27	3	72.2	25.0	2.8	183	33	84.7	15.3
rs2249210	103,948,534	T/T	T/C	C/C							
		57	38	13	52.8	35.2	12.0	152	64	70.4	29.6
rs10507172	103,949,490	G/G	G/A	A/A	G/G	G/A	A/A	G	A	G	A
		68	34	6	63.0	31.5	5.6	170	46	78.7	21.3
rs7136550	103,949,737	G/G	G/C	C/C	G/G	G/C	C/C	G	C	G	C
		67	36	5	62.0	33.3	4.6	170	46	78.7	21.3
rs10507173	103,950,058	A/A	G/A	G/G	A/A	G/A	G/G	A	G	A	G
		72	32	6	65.5	29.1	5.5	176	44	80.0	20.0
rs11111854	103,951,475	G/G	G/A	A/A	G/G	G/A	A/A	G	A	G	A
		32	53	23	29.6	49.1	21.3	117	99	54.2	45.8
rs79579452	103,952,170	T/T	T/C	C/C	T/T	T/C	C/C	T	C	T	C
		77	28	3	71.3	25.9	2.8	182	34	84.3	15.7
rs2700509	103,952,641	A/A	G/A	G/G	A/A	G/A	G/G	A	G	A	G
		50	47	11	46.3	43.5	10.2	147	69	68.1	31.9
