# Supplementary material for: Toll-like receptor chaperone HSP90B1 and the immune response to Mycobacteria
Source: PLoS One. 2018 Dec 14;13(12):e0208940. doi: 10.1371/journal.pone.0208940 (PMC6294361; doi:10.1371/journal.pone.0208940)
Supplement: Supplementary file 8 [file pone.0208940.s005.docx]

	rs2070908	rs57781511	rs1165681	rs1920413	rs3794240	rs17034943	rs1437502	rs1305392	rs1165687	rs4964142	rs2249210	rs10507172	rs7136550	rs10507173	rs11111854	rs79579452	rs2700509
rs2070908	0.44																
rs57781511	0.18	0.19															
rs1165681	0.38	0.15	0.39														
rs1920413	0.09	0.11	0.21	0.32													
rs3794240	-	0.05	-	-	0.20												
rs17034943	0.20	-	0.12	-	-	0.15											
rs1437502	-	-	-	0.10	-	0.13	0.42										
rs1305392	-	-	0.05	-	0.09	-	0.26	0.26									
rs1165687	0.07	-	0.29	-	-	-	-	0.36	0.24								
rs4964142	0.15	-	0.12	0.09	-	0.25	0.14	0.07	0.06	0.16							
rs2249210	0.09	-	0.06	-	-	0.21	0.31	0.15	0.13	0.44	0.30						
rs10507172	-	0.06	-	-	-	-	0.27	0.10	0.09	0.05	0.11	0.21					
rs7136550	0.10	-	0.13	0.10	-	-	-	0.28	0.30	0.05	0.11	0.07	0.21				
rs10507173	-	0.05	-	-	-	-	0.24	0.08	0.07	-	0.10	0.79	0.06	0.20			
rs11111854	0.13	-	0.21	0.11	-	-	0.23	0.41	0.38	0.16	-	0.23	0.32	0.19	0.46		
rs79579452	-	0.29	0.09	0.05	0.22	-	0.26	0.07	0.06	-	0.08	0.05	0.05	-	0.16	0.16	
rs2700509	0.09	-	0.16	-	0.08	-	-	0.75	0.68	0.09	0.20	0.13	0.58	0.11	0.56	0.09	0.32
