# Supplementary material for: Toll-like receptor chaperone HSP90B1 and the immune response to Mycobacteria
Source: PLoS One. 2018 Dec 14;13(12):e0208940. doi: 10.1371/journal.pone.0208940 (PMC6294361; doi:10.1371/journal.pone.0208940)
Supplement: Supplementary file 9 [file pone.0208940.s009.docx]

		CMA only						Black only					
SNP	Group	A/A	A/a	a/a	Dominant model			A/A	A/a	a/a	Dominant model		
		N (%)	N (%)	N (%)	*p*	OR	95% CI	N (%)	N (%)	N (%)	*p*	OR	95% CI
rs1920413	Control	181 (38.0)	237 (49.8)	58 (12.2)				41 (33.6)	69 (56.6)	12 (9.8)			
	TB	86 (44.1)	86 (44.1)	23 (11.8)	0.15	0.78	0.55-1.09	9 (45)	9 (45)	2 (10)	0.326	0.62	0.24-1.61
rs1305392	Control	177 (36.8)	218 (45.3)	86 (17.9)				50 (41.0)	53 (43.4)	19 (15.6)			
	TB	62 (31.5)	95 (48.2)	40 (20.3)	0.19	1.27	0.89-1.80	9 (45)	8 (40)	3 (15)	0.736	0.85	0.33-2.20
rs10507172	Control	340 (70.7)	131 (27.2)	10 (2.1)				66 (54.5)	48 (39.7)	7 (5.8)			
	TB	150 (76.1)	44 (22.3)	3 (1.5)	0.15	0.76	0.52-1.11	13 (65)	5 (25)	2 (10)	0.385	0.65	0.24-1.73
rs10507173	Control	393 (81.7)	85 (17.7)	3 (0.6)				74 (61.2)	43 (35.5)	4 (3.3)			
	TB	171 (86.8)	26 (13.2)	0 (0.0)	0.11	0.68	0.42-1.09	13 (65)	5 (25)	2 (10)	0.743	0.85	0.32-2.28
