# Supplementary material for: Toll-like receptor chaperone HSP90B1 and the immune response to Mycobacteria
Source: PLoS One. 2018 Dec 14;13(12):e0208940. doi: 10.1371/journal.pone.0208940 (PMC6294361; doi:10.1371/journal.pone.0208940)
Supplement: Supplementary file 10 [file pone.0208940.s010.docx]

SNP	Group	A/A	A/a	a/a	Dominant model		
		N (%)	N (%)	N (%)	*p*	OR	95% CI
rs1920413	Control	99 (37.4)	130 (49.1)	36 (13.6)			
	TB	59 (42.8)	64 (46.4)	15 (10.9)	0.42	0.84	0.55-1.28
rs1305392	Control	88 (33.2)	120 (45.3)	57 (21.5)			
	TB	43 (31.2)	68 (49.3)	27 (19.6)	0.8	1.06	0.67-1.67
rs10507172	Control	194 (73.2)	65 (24.5)	6 (2.3)			
	TB	106 (76.8)	29 (21)	3 (2.2)	0.45	0.83	0.51-1.35
rs10507173	Control	220 (82.7)	45 (16.9)	1 (0.4)			
	TB	121 (87.7)	17 (12.3)	0 (0)	0.2	0.67	0.36-1.23
