# Supplementary material for: Toll-like receptor chaperone HSP90B1 and the immune response to Mycobacteria
Source: PLoS One. 2018 Dec 14;13(12):e0208940. doi: 10.1371/journal.pone.0208940 (PMC6294361; doi:10.1371/journal.pone.0208940)
Supplement: Supplementary file 11 [file pone.0208940.s011.docx]

SNP	Group	A/A	A/a	a/a	Dominant model		
		N (%)	N (%)	N (%)	*p*	OR	95% CI
rs1920413	Control	193 (36.1)	278 (52.1)	63 (11.8)			
	TB	79 (44.4)	79 (44.4)	20 (11.2)	0.051	0.71	0.50-1.00
rs1305392	Control	204 (37.9)	237 (44.1)	97 (18.0)			
	TB	59 (33.0)	83 (46.4)	37 (20.7)	0.234	1.24	0.87-1.78
rs10507172	Control	356 (66.2)	166 (30.9)	16 (3.0)			
	TB	138 (77.1)	36 (20.1)	5 (2.8)	0.007	0.58	0.39-0.86
rs10507173	Control	411 (76.5)	119 (22.2)	7 (1.3)			
	TB	153 (85.5)	24 (13.4)	2 (1.1)	0.012	0.55	0.35-0.88
