# Supplementary material for: Toll-like receptor chaperone HSP90B1 and the immune response to Mycobacteria
Source: PLoS One. 2018 Dec 14;13(12):e0208940. doi: 10.1371/journal.pone.0208940 (PMC6294361; doi:10.1371/journal.pone.0208940)
Supplement: Supplementary file 12 [file pone.0208940.s012.docx]

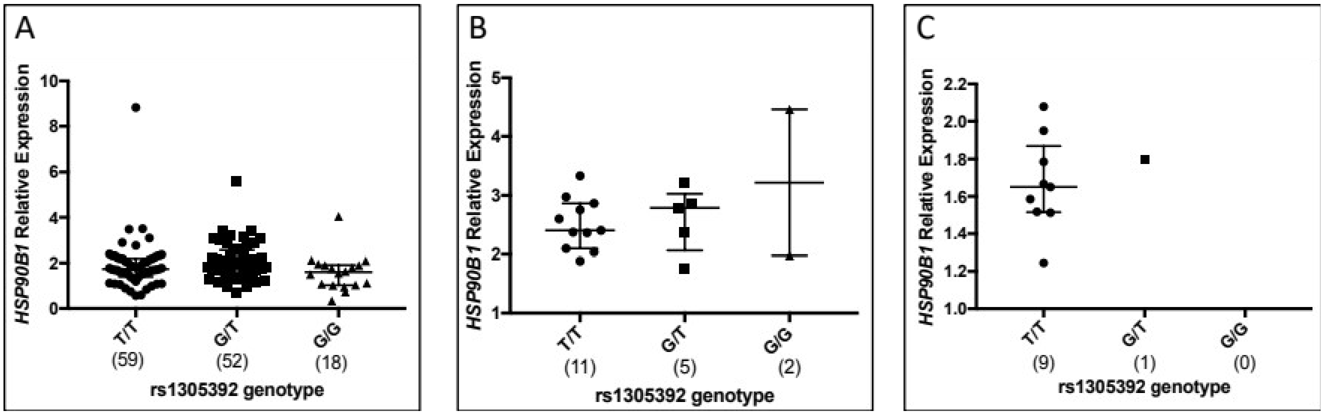
A
HSP90B1 Relative Expression
10
8
6
4
2
0
T/T
(59)
G/T
(52)
G/G
(18)
rs1305392 genotype
B
HSP90B1 Relative Expression
5
4
3
2
1
T/T
(11)
G/T
(5)
G/G
(2)
rs1305392 genotype
C
HSP90B1 Relative Expression
2.2
2.0
1.8
1.6
1.4
1.2
1.0
T/T
(9)
G/T
(1)
G/G
(0)
rs1305392 genotype
